# Complex magnetic incommensurability and electronic charge transfer through the ferroelectric transition in multiferroic Co_3_TeO_6_

**DOI:** 10.1038/s41598-017-06651-9

**Published:** 2017-07-25

**Authors:** Chi-Hung Lee, Chin-Wei Wang, Yang Zhao, Wen-Hsien Li, Jeffrey W. Lynn, A. Brooks Harris, Kirrily Rule, Hung-Duen Yang, Helmuth Berger

**Affiliations:** 1 Department of Physics, National Central University, Jhongli, 32001 Taiwan; 2 Neutron Group, National Synchrotron Radiation Research Center, Hsinchu, 30076 Taiwan; 3 NIST Center for Neutron Research, National Institute of Standards and Technology, Gaithersburg, Maryland 20899 USA; 4 Department of Materials Science and Engineering, University of Maryland, College Park, MD 20742 USA; 5 Department of Physics and Astronomy, University of Pennsylvania, Philadelphia, PA 19104 USA; 6 Bragg Institute, Australian Nuclear Science and Technology Organisation, Lucas Heights, NSW 2234 Australia; 7 Department of Physics and Center for Nanoscience and Nanotechnology, National Sun Yat-Sen University, Kaohsiung, 80424 Taiwan; 8 Institute of Physics of Complex Matter, EPFL, Lausanne, Switzerland

## Abstract

Polarized and unpolarized neutron diffractions have been carried out to investigate the nature of the magnetic structures and transitions in monoclinic Co_3_TeO_6_. As the temperature is lowered below 26 K long range order develops, which is fully incommensurate (ICM) in all three crystallographic directions. Below 19.5 K additional commensurate magnetic peaks develop, consistent with the Γ_4_ irreducible representation, along with a splitting of the ICM peaks along the *h* direction which indicates that there are two separate sets of magnetic modulation vectors. Below 18 K, this small additional magnetic incommensurability disappears, ferroelectricity develops, an additional commensurate magnetic structure consistent with Γ_3_ irreducible representation appears, and the *k* component of the ICM wave vector disappears. Synchrotron x-ray diffraction measurements demonstrate that there is a significant shift of the electronic charge distribution from the Te ions at the crystallographic 8 *f* sites to the neighboring Co and O ions. These results, together with the unusually small electric polarization, its strong magnetic field dependence, and the negative thermal expansion in all three lattice parameters, suggest this material is an antiferroelectric. Below15 K the *k* component of the ICM structure reappears, along with second-order ICM Bragg peaks, which polarized neutron data demonstrate are magnetic in origin.

## Introduction

**Figure 1:**
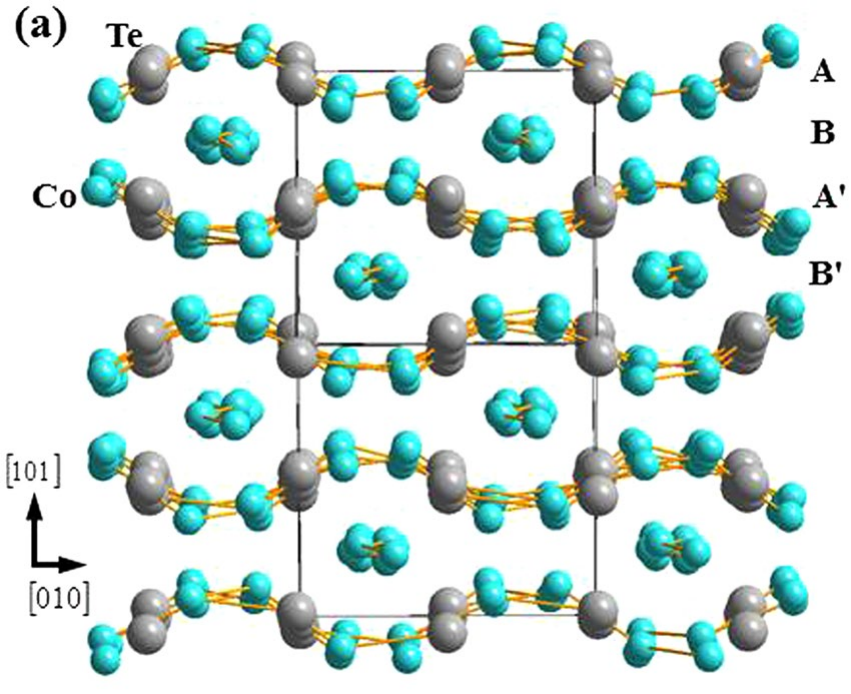
Network of the severely distorted Co/Te layers viewed along the [1, 0, −1] axis direction, without the presence of interconnecting O ions for clarity. The layers marked A′ and B′ can be reached by a translation operation of layers marked A and B, respectively, through (*a*/2, *b*/2, 0).

**Figure 2:**
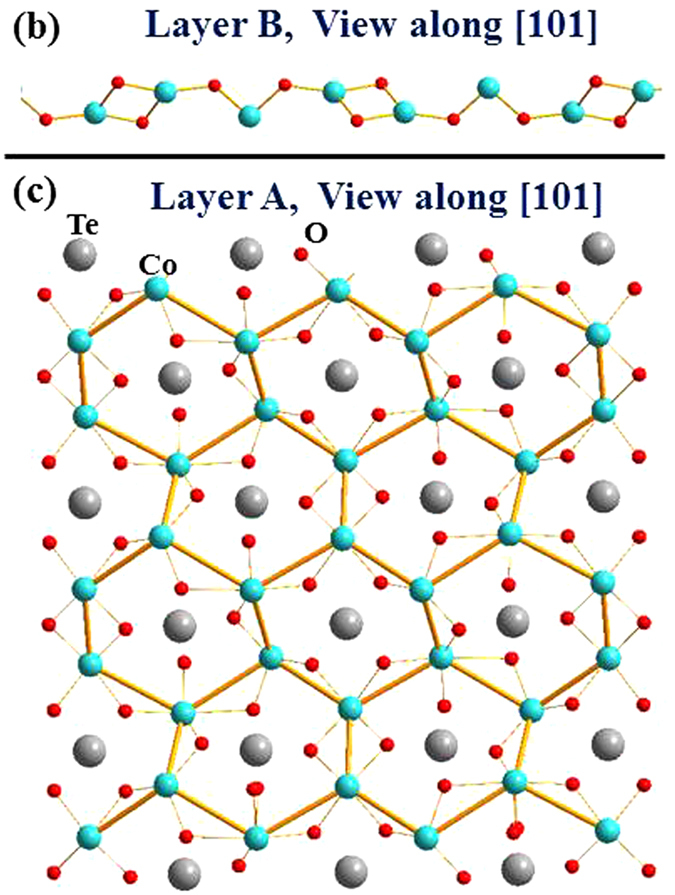
(**a**) Atomic arrangement of the Co ions in Layer B. The Co ions form well separated zigzag chains. (**b**) Atomic arrangement of the Co and Te ions in Layer A. The Co ions crystalize into a significantly distorted honeycomb, with the Te ions occupying the centers.

**Figure 3:**
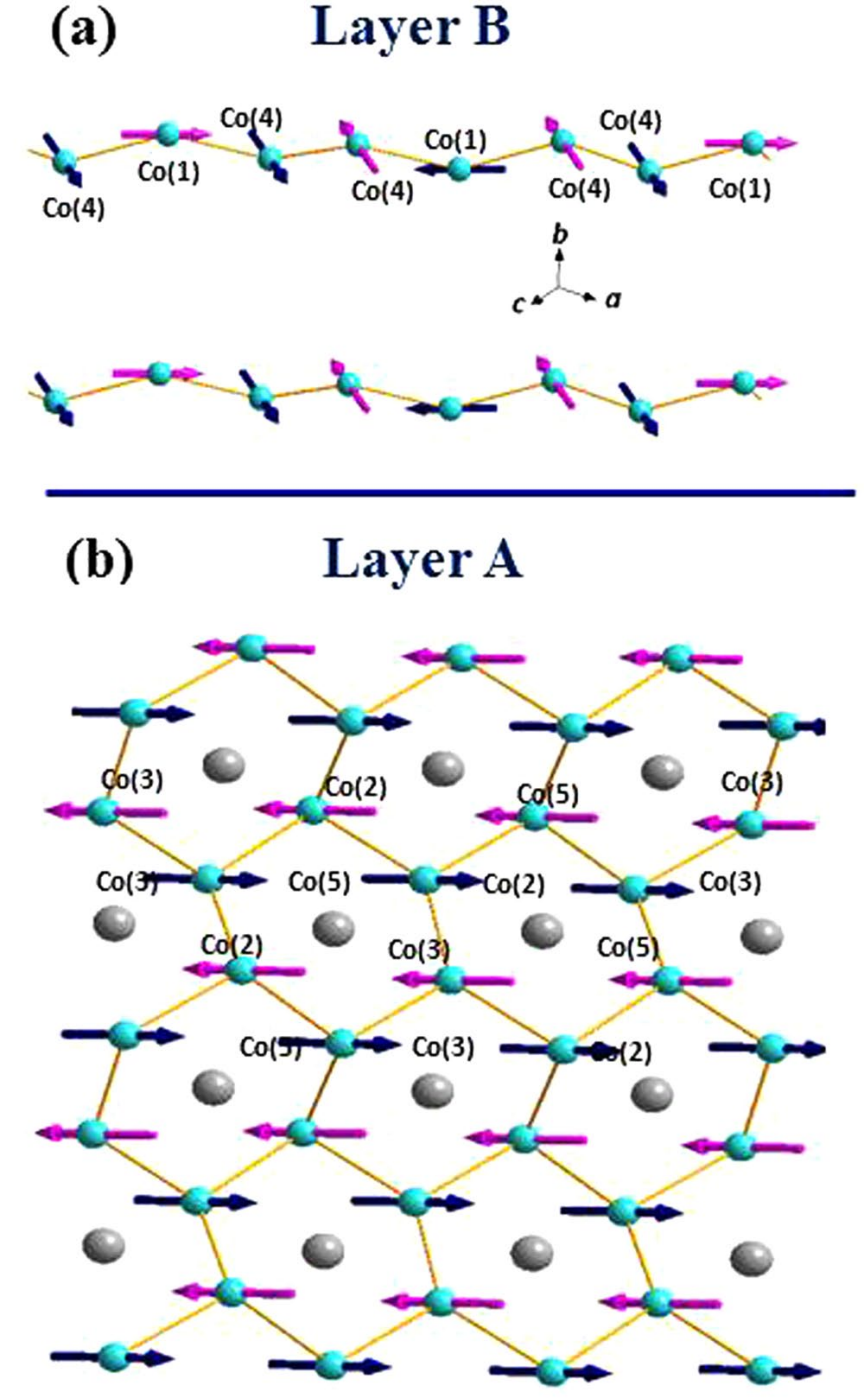
Schematic drawing of the Co spin arrangements in (**a**) Layer B and (**b**) Layer A. The Co spins in the wavy honeycomb web form a simple antiferromagnetic arrangement; whereas those in the zigzag chains form a non-collinear spin arrangement. The moments of all Co ions lie in the crystallographic *a*-*c* plane.

Many novel physical behaviors have recently been identified in the *M*_3_TeO_6_-class of metal tellurates^1^, where *M* is a first-row transition metal^2–11^. Simple commensurate (CM) magnetic structures as well as complex incommensurate (ICM) spin structures^7–11^, and spontaneous and magnetic-field-driven electrical polarizations^2, 9–11^, have all been detected. In particular, the monoclinic cobalt tellurate Co_3_TeO_6_ has been characterized^9–19^ as a type-II multiferroic, where the order parameters of electrical polarization and spontaneous magnetization are closely coupled^3, 20–27^. As the temperature is lowered, the system initially orders at T_M1_ = 26 K with a fully ICM magnetic structure, followed by a separate CM component ordering at T_M2_ = 19.5 K coexisting with the ICM magnetic order, a ferroelectric transition at T_M3_ = 18 K coupled to a change in the magnetic structures, and finally a transition at T_M4_ = 15 K into the magnetic ground state. We note that T_M4_ was defined as 16 K in our previous reports^14, 17^, while the present results reveal that this transition is better defined by 15 K, the temperature below which the *k* component of the ICM structure reappears. These transitions are naturally linked to the crystal geometry, where Co_3_TeO_6_ crystallizes into a monoclinic symmetry with the space group *C2*/*c*, with 36 Co ions in a chemical unit cell^9, 17^. The complex crystalline structure of Co_3_TeO_6_ can be viewed as being composed of six distorted Co_3_Te layers that are interconnected through O ions, forming significantly distorted corner-, edge-, and face-sharing CoO_6_ octahedra and CoO_4_ tetrahedra. The TeO_6_ octahedra are isolated from each other, but fill the spatial gaps between the CoO_6_ octahedra. There are five crystallographically distinct Co ions that occupy two very different layered arrangements (Fig. 1)^9, 17^. One is comprised of distorted zig-zag Co-O-Co chains (marked Layer B, Fig. 2a) which mediate antiferromagnetic superexchange (SE) interactions for the Co spins. The Co ions in the other separate layers (marked Layer A, Fig. 2b) form a severely distorted honeycomb web, with the Te ions located in the channels. The Co-O bond lengths and Co-O-Co bond angles in both Layer A and Layer B vary widely. Their differences can be as large as 50%, which result in widely varying magnetic interactions that compete within the unit cell. The spin structure at 3 K has been reported^17^, where the Co spins in the zig-zag chains in Layer B order into a complex ICM spin arrangement (Fig. 3), with a modulation vector of ***q*** = (0.357, 0.103, 0.087) for the magnetic propagation vector. In contrast to the previous data taken in a single scattering plane^14^, the present experiments demonstrate that all three components of the ordering wave vector are incommensurate, along with second-order ICM Bragg peaks that are also magnetic. The Co spins in Layer A, on the other hand, develop a simple CM antiferromagnetic order with Γ_4_ symmetry at zero wave vector. The non-collinear spin arrangement together with the appearance of ICM components for the Co spins in the well-separated zig-zag chains in Layer B show that they experience more complicated magnetic couplings than the Co spins in Layer A. This is linked to the Co-Co separations in the zig-zag chains being significantly shorter (3.30 Å in average), so that the secondary interactions are also evidently revealed in addition to the indirect Co-O-Co SE interactions. The delicate balance of the magnetic interactions with temperature generates a cascade of magnetic transitions coupled to the lattice.

In this study we focus on new aspects of this interesting but complicated multiferroic, including the magnetic structure, charge density, and coupling to the ferroelectric order. The evolution of the ICM propagation vector with temperature reveals that all three (*h*, *k* and *l*) reciprocal lattice directions exhibit incommensurability. In addition, we demonstrate directly that the intensity map of the *k*-*l* scattering plane excludes having CM ordering at the (0 1/2 1/4) wave vector, suggested in a report^9^ based on early results from a powder sample and profile refinement. We also observe second-order ICM reflections that develop below 15 K, that polarized neutrons demonstrate are magnetic in nature rather than structural. Finally, synchrotron powder x-ray diffraction data show that for the Te ions at the crystallographic 8 *f* sites a significant amount of the electric charge shifts from the Te ions to their neighboring Co and O ions upon cooling through the ferroelectric transition. This finding is significant because the spontaneous polarization is very small for a ferroelectric, and in this case may indicate that the ferroelectricity is associated with some type of larger antiferroelectric order parameter as we discuss later.

## Materials and Methods

Single crystals of Co_3_TeO_6_ were synthesized through chemical vapor transport redox reactions as described previously^14, 17^. The single crystal used in the present measurements was the same as in our previous work, and weighed 101 mg with a size of 14.1 × 2.2 × 0.9 mm^3^. A number of small crystals were crushed into powder for x-ray powder diffraction measurements. X-ray diffraction was first used to check the powdered sample. No obvious differences were found in x-ray diffraction patterns taken from different portions of the powdered sample. Details of the sample fabrication and characterization can be found in the Supplementary Information. Although the thermal magnetization of Co_3_TeO_6_ has been discussed in detail^2, 9, 13, 18, 19^ and the results are not a part of the principal findings that are communicating, the thermal variations of ac magnetic susceptibility, for the purpose of completeness, are provided in the Supplementary Information as well.

Single crystal neutron diffraction measurements were conducted at the NIST Center for Neutron Research using the BT-7 triple-axis spectrometer with λ = 2.359 Å defined by PG (002) crystals at both the monochromator and analyzer positions, or a position-sensitive-detector (PSD) without energy analysis^28^. Data were also collected at the Bragg Institute, ANSTO, using the thermal triple-axis spectrometer Taipan [λ = 2.35 Å defined by PG (002) crystals at both the monochromator and analyzer positions]. The polarized neutron-diffraction measurements were conducted on BT-7, using ^3^He polarizers placed both before and after the sample position for polarization analysis^29^. The neutron polarization direction was realized by a guide field along (horizontal H-field) or perpendicular (vertical V-field) to the scattering plane. The single crystal was mounted to allow access to the (*hk0*), (*h0l*) or (*0kl*) crystallographic plane for a complete *h*-*k*-*l* reciprocal space scattering intensity survey. One point to keep in mind about the neutron measurements is that the instruments have quite coarse vertical resolution, which integrates the scattering perpendicular to the scattering plane. For incommensurate peaks that are displaced not too far away from the scattering plane this still allows their observation by projecting their intensity onto the scattering plane. If one has a reflection at an arbitrary wave vector ***q***_**1**_ = (*h*, *k*, *l*), then in view of the *C2/c* symmetry of the crystal one expects reflections at ***q***_***i***_ with *i* = 1~4, where ***q***_**1**,**2**_ =  ± (*h*, *k*, *l*) and ***q***_**3**,**4**_ = ±(*h*, −*k*, *l*). We observe such reflections by monitoring the scattering in one or more of the *h* = 0, *k* = 0, or *l* = 0 planes. In the *h* = 0 scattering plane, for example, the vertical resolution is coarse enough to integrate the ICM’s at nonzero *h*. We refer to such a spectrum as a “projected” scan and indicate which coordinate is integrated. Measurements in several projections then allows a complete determination of the components of the ICM wave vector, which also have been verified by data taken with tighter vertical resolution, as discussed in more detail below.

The high-resolution synchrotron x-ray powder diffraction patterns were collected on the BL01C2 beam line at the Taiwan National Synchrotron Radiation Research Center, employing an incident wavelength of 0.77495 Å as defined by a Si(111) double-crystal monochromator. The diffraction intensities were recorded by a Mar345 imaging plate system, with a sample-to-detector distance of 300 mm. The sample temperature was controlled using a He-gas closed-cycle refrigerator system, equipped with a high power heater. Throughout the paper, uncertainties where indicated represent one standard deviation.

## Results and Discussion

### Magnetic phases

**Figure 4:**
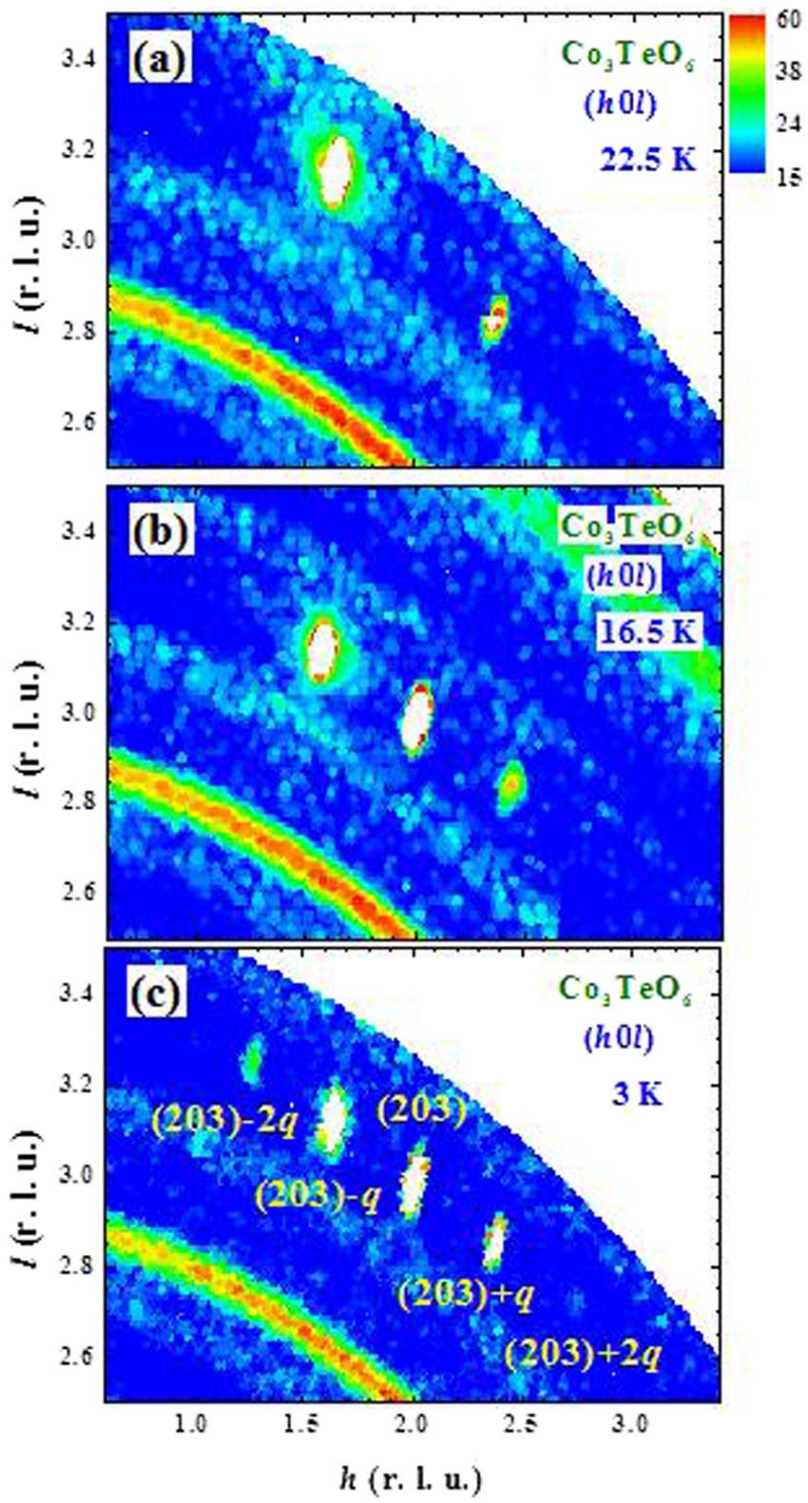
Neutron-diffraction intensity maps in the (*h*0*l*) scattering plane near the (203) Bragg position observed at (**a**) 22.5 K, (**b**) 16.5 K, and (**c**) 3 K. The strong powder ring is from the Al sample holder. Below the initial ordering at T_M1_ = 26 K there is no detectable intensity at the commensurate (203) Bragg position at 22.5 K (**a**), but two satellite magnetic reflections are revealed arising from the development of incommensurate magnetic order. We remark that the incommensurability occurs in all three components of the wave vector, but in this scattering plane the larger vertical divergence of the instrument integrates of the k components. (**b**) Below T_M2_ = 19.5 K the commensurate (203) magnetic reflection (and equivalent CM peaks) appears, accompanied by two satellite magnetic reflections at (203) ± (*q*_*h*_, *q*_*k*_, *q*_*l*_). (**c**) In the ground state (3 K), in addition to the two first-order satellites, two second-order satellite magnetic reflections at the (203) ± 2(*q*_*h*_, *q*_*k*_, *q*_*l*_) positions are also visible.

For the *C2*/*c* crystalline symmetry of monoclinic Co_3_TeO_6_ the nuclear (203) Bragg peak is forbidden, making it an excellent place to investigate the magnetic order. In the (*h*, 0, *l*) scattering plane we see the development of two ICM satellite reflections at the (2 − *q*_*h*_, ±*q*_*k*_, 3 + *q*_*l*_) and (2 + *q*_*h*_, ±*q*_*k*_, 3 − *q*_*l*_) positions with *q*_*h*_ = 0.365(1) and *q*_*l*_ = 0.106(1), as shown in Fig. 4a for T = 22.5 K. We remark that the incommensurability occurs in all three components of the wave vector as we discuss in detail below. Scans of these peaks in the vertical direction were made with the monochromator in vertical focusing mode, and then there barely is any indication of a splitting, but the peaks could readily be resolved using a vertically flat monochromator. It appears that the intensity of the (203) − ***q*** reflection is 5.1 times higher than that of the (203) + ***q*** reflection. This noticeable difference in intensity of the two satellites in this (Fig. 4a) scattering plane is likely due to the orientation factor in the magnetic cross section. Note that there is no magnetic intensity at the CM (203) position (or any other commensurate magnetic reflection) at this temperature, which peaks first appear below T_M2_ = 19.5 K. In the ferroelectric phase at 16.5 K the *q*_*h*_ of the ICM satellite reflections shifts to a larger value (Fig. 4b), while further cooling into the ground state increases the magnetic intensities as usual, while both *q*_*h*_ and *q*_*l*_ shift back to smaller values (Fig. 4c). More interesting is the development of second-order ICM satellite reflections at the (2 − 2*q*_*h*_, ±2*q*_*k*_, 3 + 2*q*_*l*_) and (2 + 2*q*_*h*_, ±2*q*_*k*_, 3 − 2*q*_*l*_) positions as clearly visible in the 3 K intensity map, which were also investigated with detailed vertical scans. Typically, in the case of a ICM spin-density wave second-order peaks reflect a charge-density-wave (magnetostructural coupling) accompanying the spin-density-wave, since if there’s no breaking of the AFM symmetry (no net magnetization) only higher-order reflections that are odd numbers are allowed. As we will see below, however, these second-order peaks are magnetic, which indicates a small net magnetization has developed below T_M4_, as indicated by Toledano, *et al*.^16^ and later by Singh, *et al*.^19^, similar to what has been found in ErNi_2_B_2_C^30^, for example. Apparently, the Co spins in Layer A and in Layer B order separately, reflecting a weak interlayer magnetic coupling for Co_3_TeO_6_. The two magnetic transitions found^2, 9, 13, 17^ are the result of the ordering of the Co spins in the zigzag chains in Layer B into an ICM spin arrangement at T_M1_ = 26 K, and the separate ordering of the Co spins in the honeycomb web in Layer A into a CM spin structure at T_M2_ = 19.5 K. Clearly, the modulation vector ***q*** = (*q*_*h*_, *q*_*k*_, *q*_*l*_) of the satellite reflections is very sensitive to temperature as previously discussed^14^.

**Figure 5:**
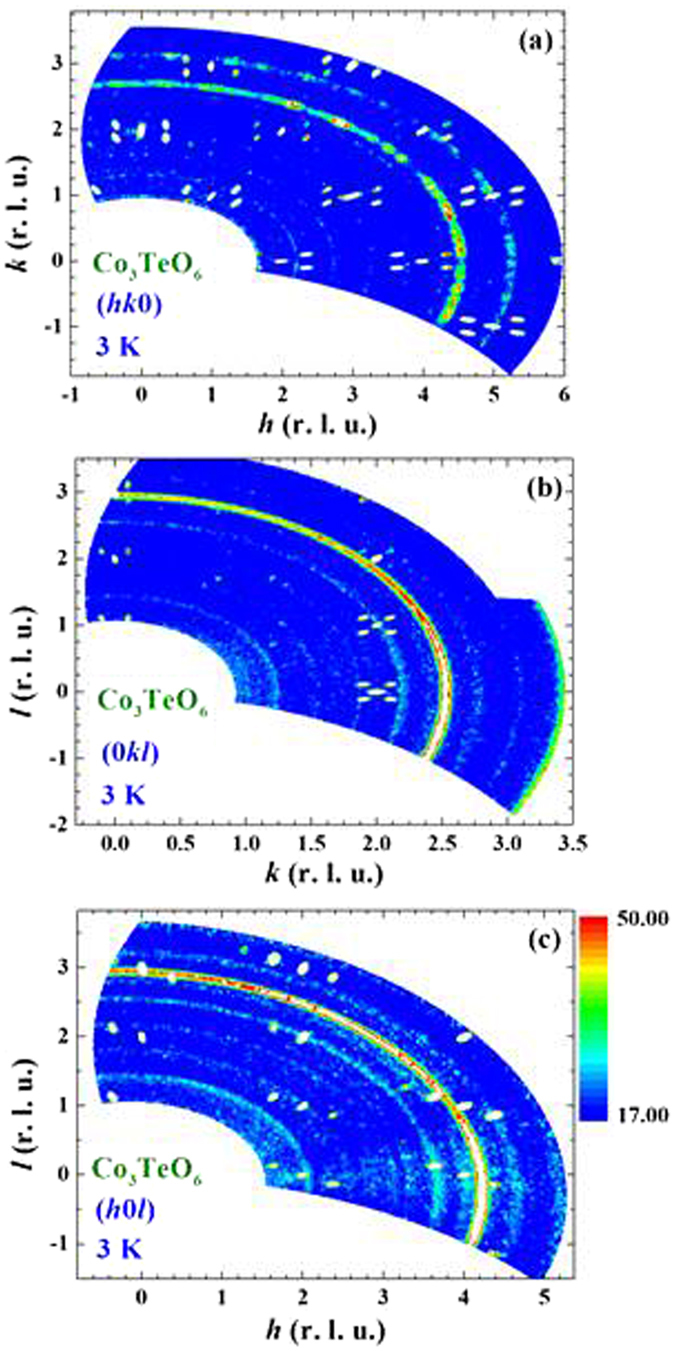
Neutron-diffraction intensity maps observed in the (**a**) (*hk0*), (**b**) (*0kl*) and (**c**) (*h0l*) scattering planes, obtained at 3 K, using unpolarized neutrons. Four satellite magnetic reflections associated with each commensurate Bragg position are revealed in the (*hk0*) and (*0kl*) scattering planes; while two satellite magnetic reflections are revealed for each reflection in the (*h0l*) scattering plane. Second-order satellite magnetic reflections associated with the reflections at the (203), (400) and (401) Bragg positions are evident.

The intensity maps at 3 K of the (*hk0*), (*0kl*) and (*h0l*) scattering planes are shown in Fig. 5. Four ICM satellite components associated with each Bragg peak are seen for the reflections in the (*hk0*) (Fig. 5a) and (*0kl*) (Fig. 5b) scattering planes. Interestingly, there are only two ICM components associated with each Bragg peak revealed in the (*h0l*) scattering plane with the modulation only in the +*q*_*h*_ or in the +*q*_*l*_ component (Fig. 5c). The modulation vector is determined to be ***q*** = (0.357, 0.103, 0.122) at 3 K. Four sets of second order ICM reflections associated with the (020), (110), (203) and (400) Bragg reflections are also observed (Fig. 5a and c). The appearance of four-**q** ICM reflections in the (*hk0*) and (*0kl*) scattering planes but two-**q** in the (*h0l*) scattering plane are the direct result of the existence of four modulation vectors that are associated with each CM Bragg reflection, driven by the *C/2c* crystalline symmetry. As illustrated in Fig. S3 in the Supplementary Information, the four ICM reflections at (+*q*_*h*_, 2 + *q*_*k*_, −*q*_*l*_), (+*q*_*h*_, 2 − *q*_*k*_, −*q*_*l*_), (−*q*_*h*_, 2 + *q*_*k*_, +*q*_*l*_) and (−*q*_*h*_, 2 − *q*_*k*_, +*q*_*l*_) (crosses) will be detected by the instrumental resolution at four positions (solid circles) upon scanning in the (*hk0*) scattering plane, but will be detected at only two positions when is scanning in the (*h0l*) scattering plane.

The neutron polarization analysis technique can be employed to unambiguously establish if these incommensurate peaks have a magnetic or structural origin^31, 32^. Nuclear coherent Bragg scattering never causes a reversal, or spin-flip, of the neutron spin direction upon scattering, while the magnetic cross-sections depend on the relative orientation of the neutron polarization **P** and the reciprocal lattice vector **Q**. In the configuration where **P||Q**, all the magnetic scattering is spin-flip. The arrangement of measuring both **P||Q** and **P**⊥**Q** then provides an unambiguous method of distinguishing magnetic from structural Bragg peaks.

**Figure 6:**
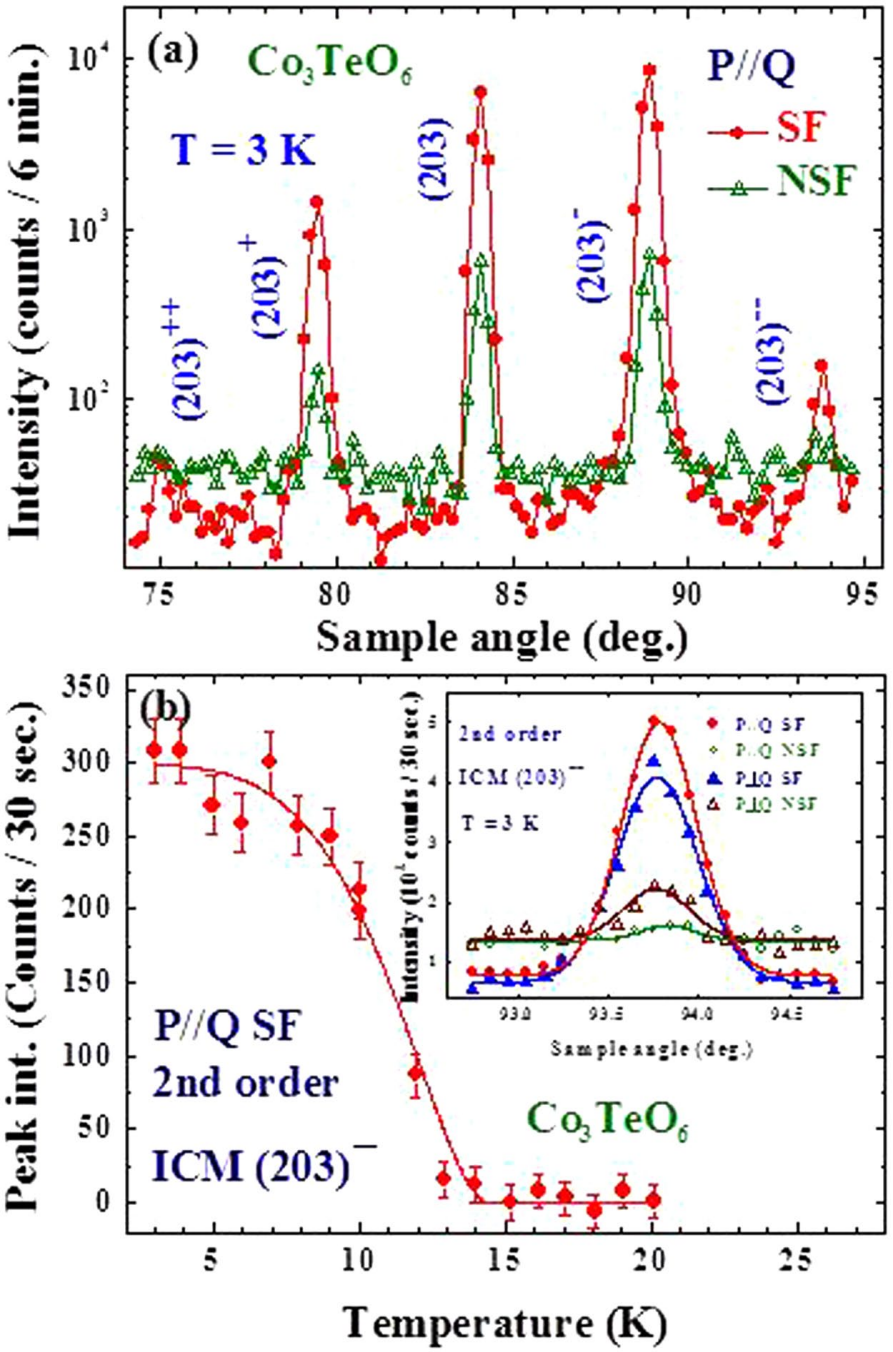
Polarized neutron diffraction measurements for the commensurate and incommensurate Bragg reflections. (**a**) Direct comparison of the spin-flip (SF, filled circles) and non-spin-flip (NSF, open triangles) scans at 3 K, covering two first-order and two second-order satellite reflections associated with the (203) CM magnetic Bragg reflection. Intensity differences between the SF and NSF channels for the five peaks are clearly revealed, showing that all five peaks are magnetic in origin. (**b**) Temperature dependence of the peak intensity of the second-order satellite (203) − 2(*q*_*h*_, 0, *q*_*l*_) reflection, revealing that these second-order peaks only develop significant intensity below ≈15 K (T_M4_). The solid curve is an order-parameter fit to the data to provide a simple guide-to-the-eye. The inset shows the (203) − 2(*q*_*h*_, 0, *q*_*l*_) reflection taken with four polarization channels.

The magnetic origin of the CM (203) Bragg reflection and the associated ICM satellite reflections at 3 K is confirmed by the polarized neutron measurements as shown in Fig. 6a, where intense spin-flip (SF) scattering from (203), (203) ± ***q*** and even from (203) ± 2***q*** are revealed. The small peaks that appear in the non-spin-flip (NSF) channel are due to imperfect polarization of the incident beam, rather than from nuclear diffraction. Significant reduction of the intensity of the second order (203) − 2***q*** ICM reflection appears above 9 K, which becomes barely visible above ≈13 K (Fig. 6b), while this series of first-order ICM reflections persist up to 26 K. We associate the development of these second-order peaks with the transition T_M4_ into the ground state spin configuration.

**Figure 7:**
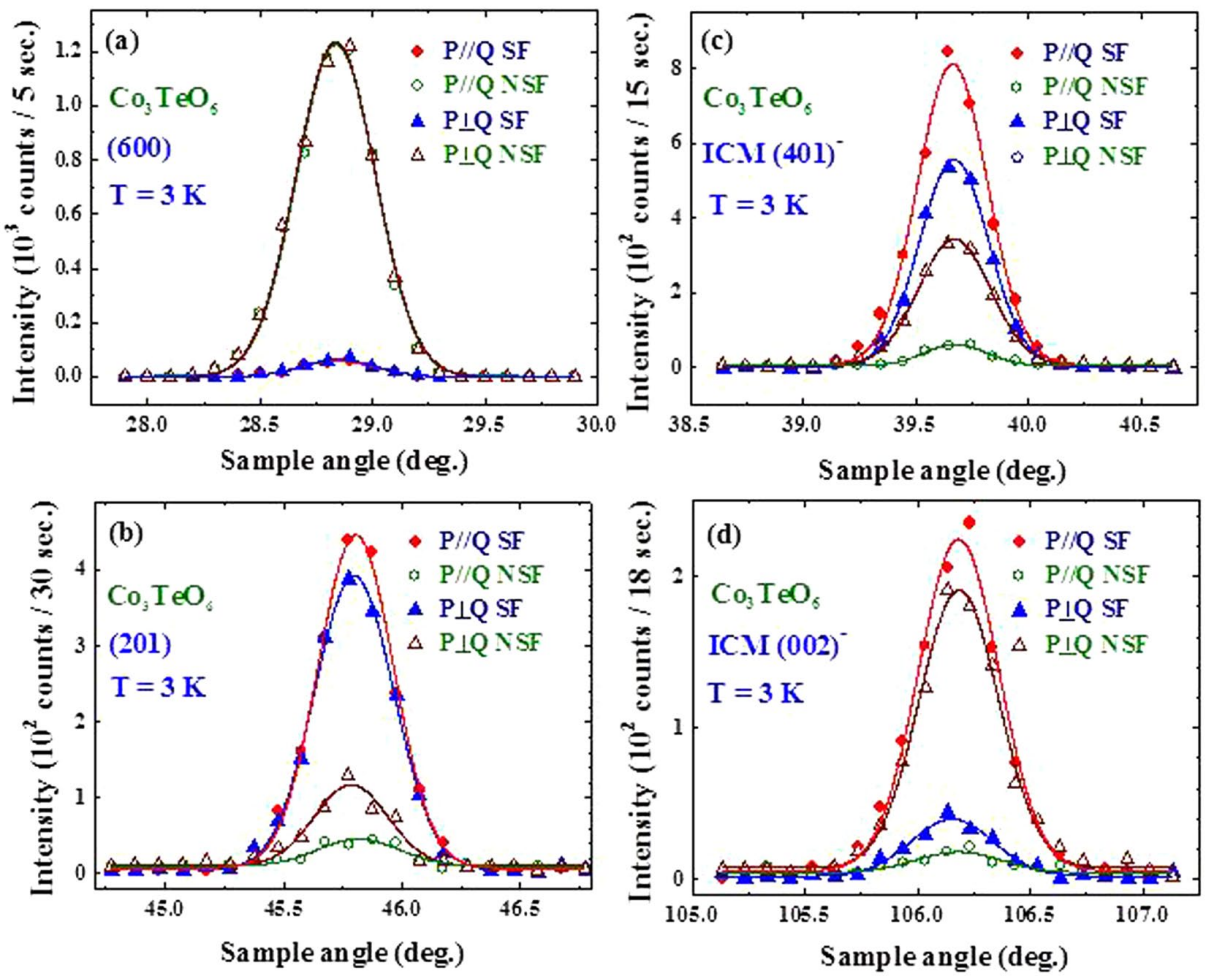
Polarized neutron diffraction data for spin-flip and non-spin-flip intensities at 3 K in horizontal and vertical field configurations for: (**a**) the (600) Bragg reflection; (**b**) (201) Bragg reflection; (**c**) (401)^−^ satellite reflection; and (**d**) (002)^−^ satellite reflection. For the (600) peak only non-spin-flip scattering is observed (above the instrumental flipping ratio), independent of guide field direction, demonstrating that this peak is purely nuclear and has no magnetic component. Magnetic components are observed for the other three reflections.

**Figure 8:**
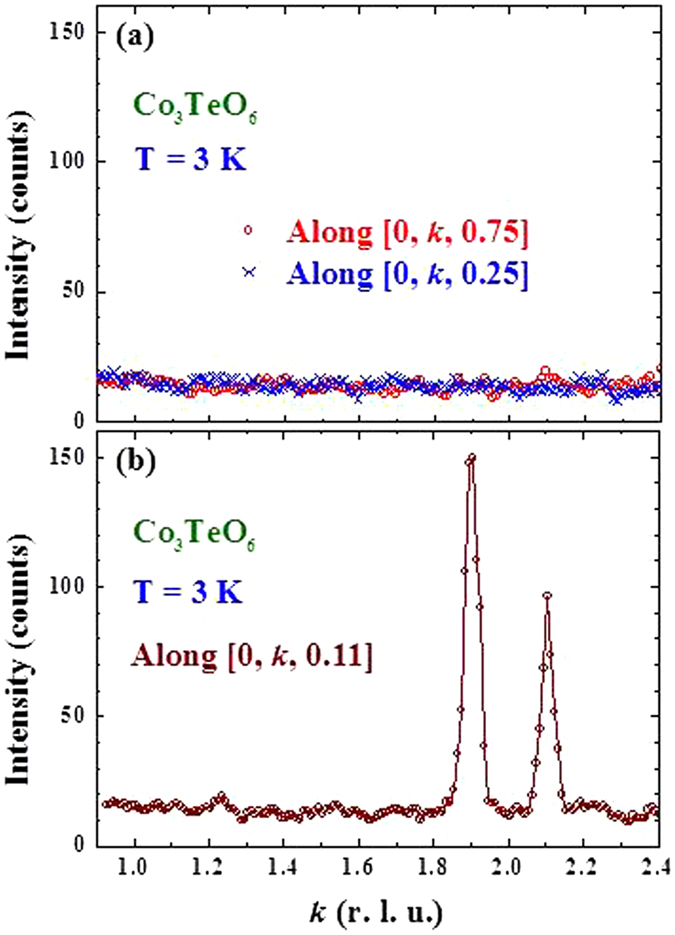
(**a**) Scans along the [0, k, 0.25] (crosses) and [0, k, 0.75] (open circles) crystallographic directions at 3 K, compared with the first-order incommensurate magnetic peaks along [0, k, 0.11] at the (0, 1.905, 0.11) and (0, 2.095, 0.11) positions, on the same intensity scales for direct comparison. No magnetic intensities are detected at the (0, 0.5, 0.25) or (0, 0.5, 0.75) positions, demonstrating that such peaks are not associated with the Co_3_TeO_6_ magnetic structure.

Figure 7a reveals that there is no magnetic component of the (600) Bragg reflection even at 3 K, which is consistent with the magnetic structure proposed^17^ based on powder diffraction results. The intensities appear in the SF channel reveal a 95% polarization for the incident beam. We remark that the change in the (600) Bragg intensity of this single crystal below 16 K reported in ref. 14 originates from the relief of extinction in this high quality crystal as discussed previously (shown in Fig. 10b of ref. 17). Other structural Bragg peaks, such as the (201) Bragg reflection (Fig. 7b), do develop magnetic contributions. In the scattering geometry with **P** perpendicular to **Q** the magnetic scattering from ordered moments with components along **P** is entirely non-spin-flip. The large intensities revealed in the **P**⊥**Q** SF channel of the (203) − 2***q*** (inset to Fig. 6b), (401) − ***q*** (Fig. 7c), and (002) − ***q*** (Fig. 7d) reflections demonstrate the departure of the ICM spin structure from collinearity in the crystallographic *a*-*c* plane. One important point that needs to be addressed is the report, based on early powder diffraction analysis^9^, of a commensurate (0, 0.5,﻿ 0.25)-type of reflection. The (*0kl*) intensity map shown in Fig. 5b indicates that there is no intensity associated with this type of reflection. Figure 8 shows a direct comparison of the incommensurate satellite peaks in Fig. 8b with scans in Fig. 8a that should show this (0, 0.5, 0.25)-type of reflection. The absence of any intensity is a direct demonstration that the (0, 0.5, 0.25) propagation vector is not in fact present in this system.

### ICM order and splitting of the modulation vector

**Figure 9:**
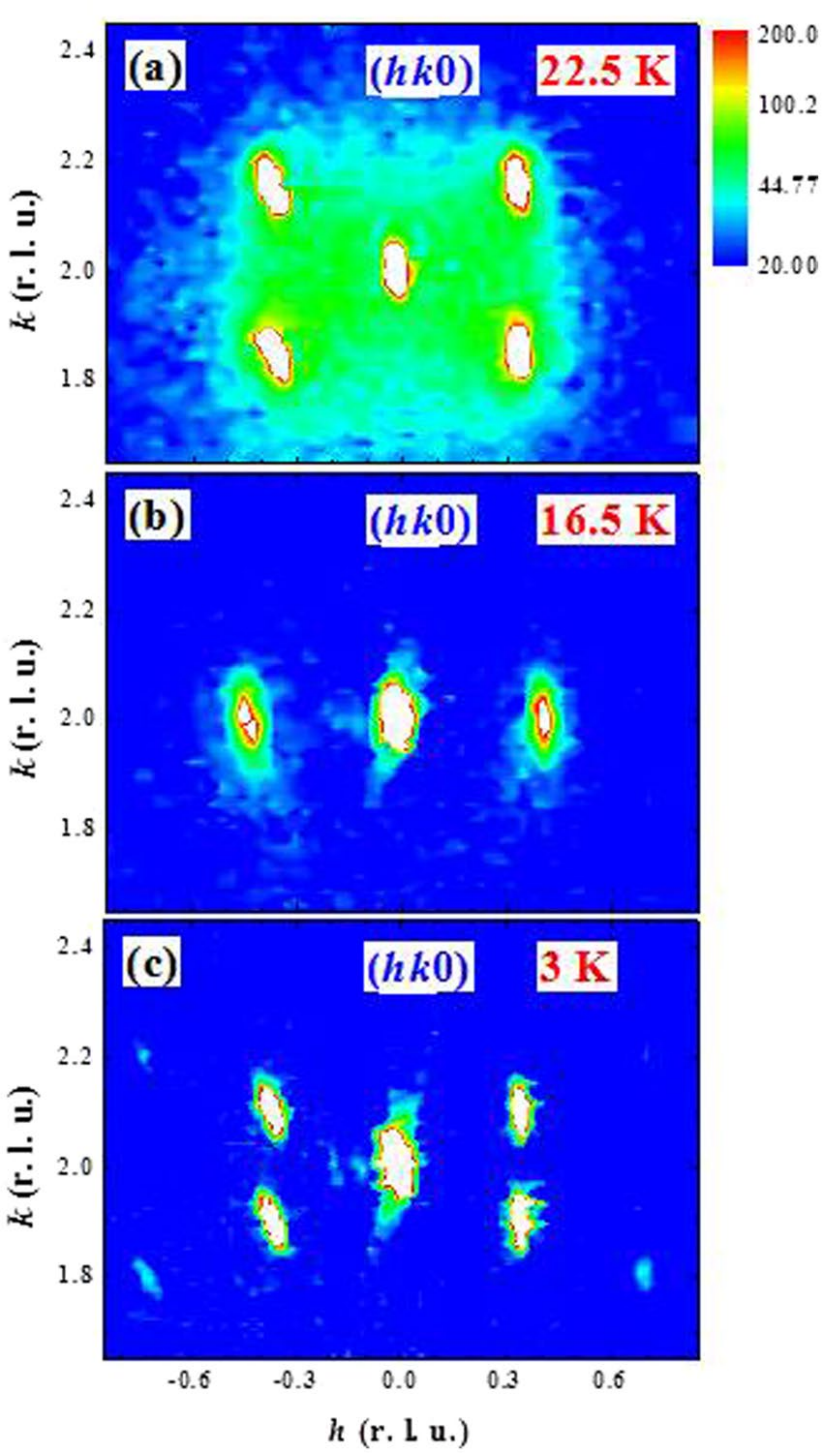
Neutron-diffraction intensity maps in the (*hk0*) scattering plane near the (020) Bragg position observed at (**a**) 22.5 K, (**b**) 16.5 K and (**c**) 3 K. Four satellite magnetic reflections appear at 22.5 K, but the incommensurability along the *h* direction vanishes at 16.5 K, demonstrating the occurrence of a magnetic phase transition. The incommensurability along the *k* direction is re-established below T_M4_, demonstrating the occurrence of another magnetic phase transition. Second-order peaks are also observed in the ground state.

Both the intensities and wave vector of the ICM spin structure, which are known to be associated with the Co spins in the zigzag chains, vary strongly with temperature, as readily revealed by examining the ICM peaks around the strongest (020) + ***q*** series. Below the initial ordering temperature of T_M1_ = 26 K we have four satellite reflections at (020) + ***q***_***i***_, with ***q***_**1**,**2**_ =  ± (*q*_*h*_, *q*_*k*_, -*q*_*l*_) and ***q***_**3**,**4**_ = ± (*q*_*h*_, −*q*_*k*_, −*q*_*l*_) with *q*_*h*_ = 0.356 and *q*_*k*_ = 0.155 at 22.5 K (Fig. 9a). We also note diffuse intensity around and between the peaks at this temperature near T_M1_, which is inelastic (critical) scattering since these data close to the initial ordering temperature were taken using the PSD without an energy analyzer. In the ferroelectric phase at 16.5 K the four peaks have collapsed into two, with *q*_*k*_ = 0, while *q*_*h*_ shifts to a larger value as shown in Fig. 9b. In the ground state (T = 3 K) the *k* component of the incommensurability is restored. Interestingly, we observe four second-order ICM reflections at (020) + 2***q***_***i***_ (Fig. 9c), indicating that the ICM magnetic structure has distorted and is no longer described by a simple sinusoid.

**Figure 10:**
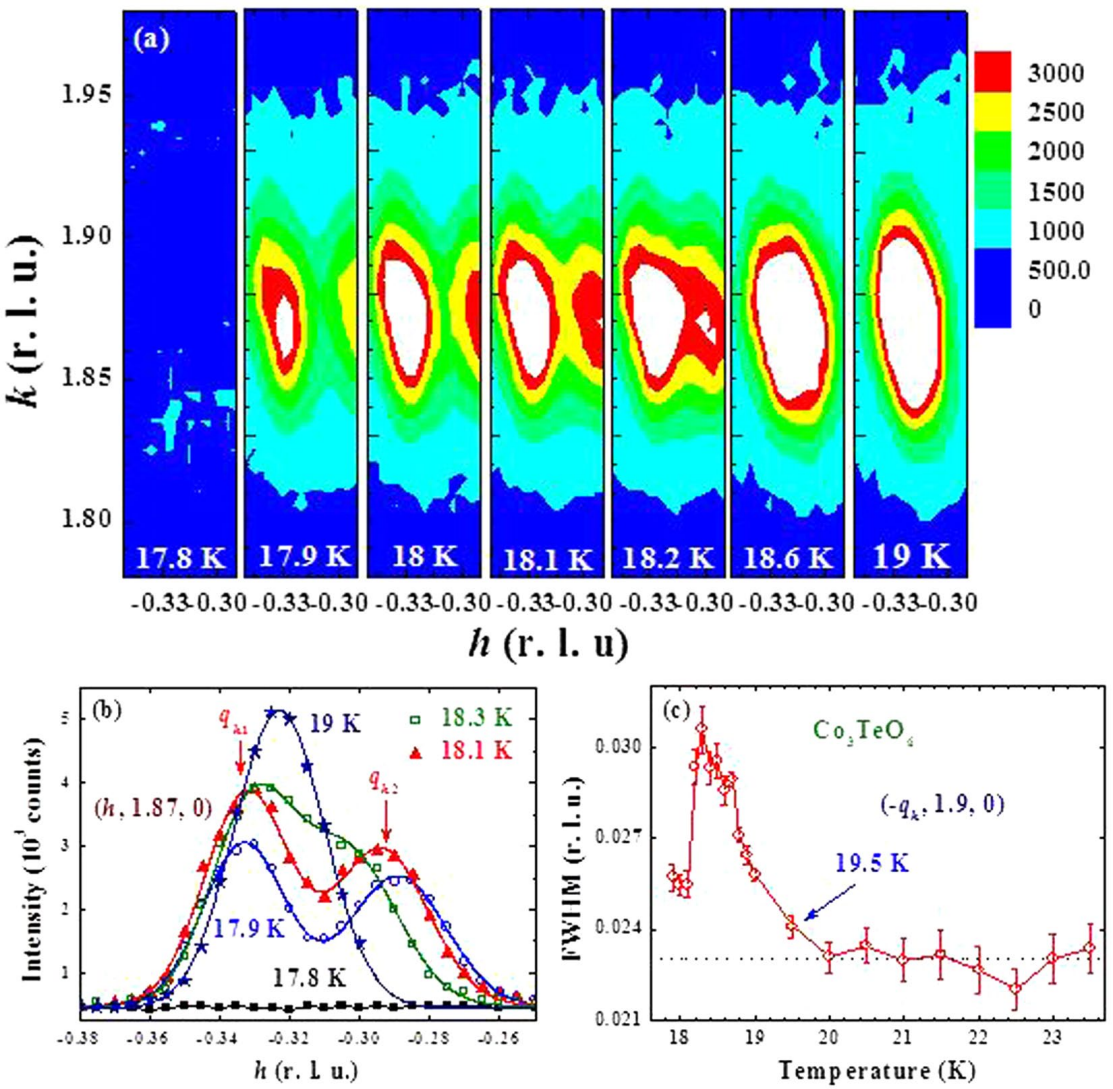
(**a**) Thermal variations of the intensity maps of the (020)^−^ satellite magnetic reflection, covering 17.8 to 19 K. Splitting of the satellite reflection in the h direction is clearly seen on cooling below 18.2 K. (**b**) Diffraction peak profiles of the satellite (020)^−^ reflection taken along the *h* direction at five representative temperatures, revealing the splitting of the reflection. (**c**) Temperature dependence of the full-width-at-half-maximum (FWHM) of one of the satellite reflection around (020).

A detailed examination of the ICM peaks with higher instrumental resolution indicates a remarkable new feature. Below 20 K the width of the reflection increases in *q*_*h*_ when cooling below 20 K (Fig. 10c), suggesting that the single peak structure observed between 18.6 and 19.5 K actually contains two inseparable peaks. Upon further cooling the peak clearly splits into two as shown in Fig. 10a. At 18.6 K we have one peak located at *q*_*h*_ = 0.310(1), and we see that the peak splits asymmetrically, with the reflection at smaller *q*_*h*_ shifting ≈3.6 times more than that of the peak that is shifting to a larger *q*_*h*_ value as shown in Fig. 10b. The two ICM reflections appear in the (*hk0*) scattering plane at *q*_*h*1_ = 0.344(1) and *q*_*h*2_ = 0.289(1) at 17.9 K, prior to moving away from this scattering plane at 17.8 K.

**Figure 11:**
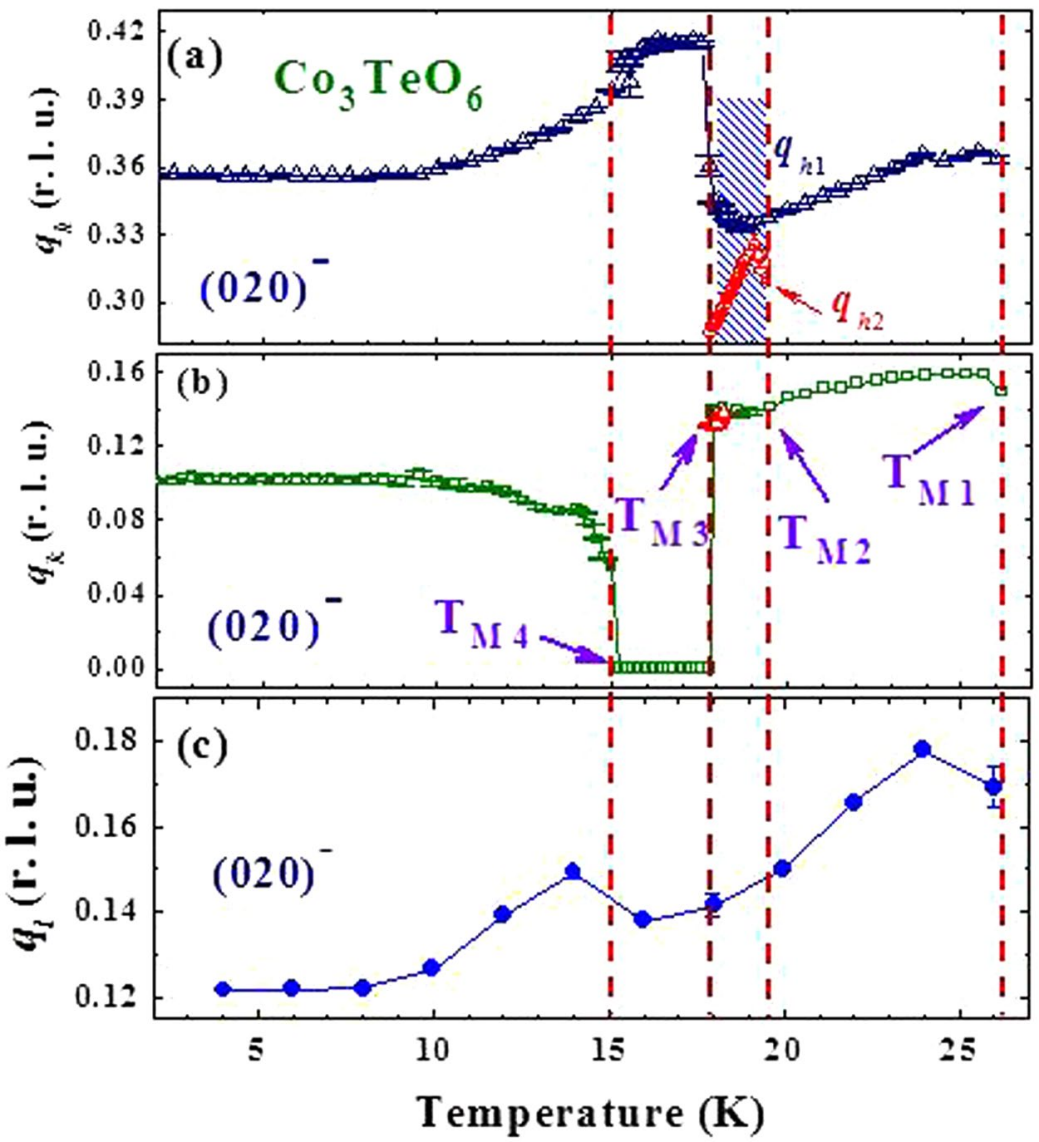
Thermal variations of (**a**) *q*_*h*_ (**b**) *q*_*k*_ and (**c**) *q*_*l*_ of the (020)^−^ satellite reflection, revealing all three components of the modulation vector change with temperature. The modulation vector stabilizes below 10 K, where the second-order satellite intensities become fully developed. There are two sets of *q*_*h*_, marked *q*_*h1*_ and *q*_*h2*_, which appear between 18 and 19.5 K. The dashed vertical lines indicate the temperatures at which the transitions are observed in Co_3_TeO_6_.

All three components of the magnetic ICM modulation vector vary substantially with temperature, with *q*_*h*_ ranging from 0.334 to 0.417, *q*_*k*_ from 0 to 0.159 and *q*_*l*_ from 0.122 to 0.178 as shown in detail in Fig. 11. The modulation vector stabilizes below ≈10 K, which is about where the second-order satellites develop their full intensities. Interestingly, the two-*q*_*h*_ structure begins to appear below T_M2_, which is the temperature at which antiferromagnetic ordering of the Co spins in Layer A begins to develop^17^. The two-*q*_*h*_ structure disappears below 18 K, which is the temperature below which ferroelectricity develops. Note that all three components of the modulation vector remain essentially unaltered between 16 and 18 K during the development of ferroelectricity, and there is a discontinuous jump in *q*_*h*1_ at 18 K when links to the ICM spin structure of the Co spins in Layer B that develops below T_M1_ = 26 K. The *q*_*h*2_ branch, on the other hand, is separate from the *q*_*h*_’s observed at other temperatures, and its origin is not clear. One possibility is that the ICM order on the chains couples to the honeycomb Layer A, and the competing interactions result in a different, weaker ICM order in this layer as it begins to order spontaneously. Alternatively, it could represent a second type of modulation on Layer B. In any event, this modulation suddenly disappears at the ferroelectric transition, where the *q*_*k*_ component suddenly locks in to zero, and the magnetic intensity in the commensurate Layer B order jumps in amplitude at this first order transition. Clearly the ferroelectricity is connected to the magnetic order on both sublattices, but appears to be more strongly influenced by the Co spins in Layer A since this is the magnetic symmetry that allows the ferroelectric order, and is the one where the applied electric field noticeably enhances the intensity of the commensurate magnetic reflections (Figs 8, 9 and 11 of ref. 17).

### Electronic charge redistribution

**Figure 12:**
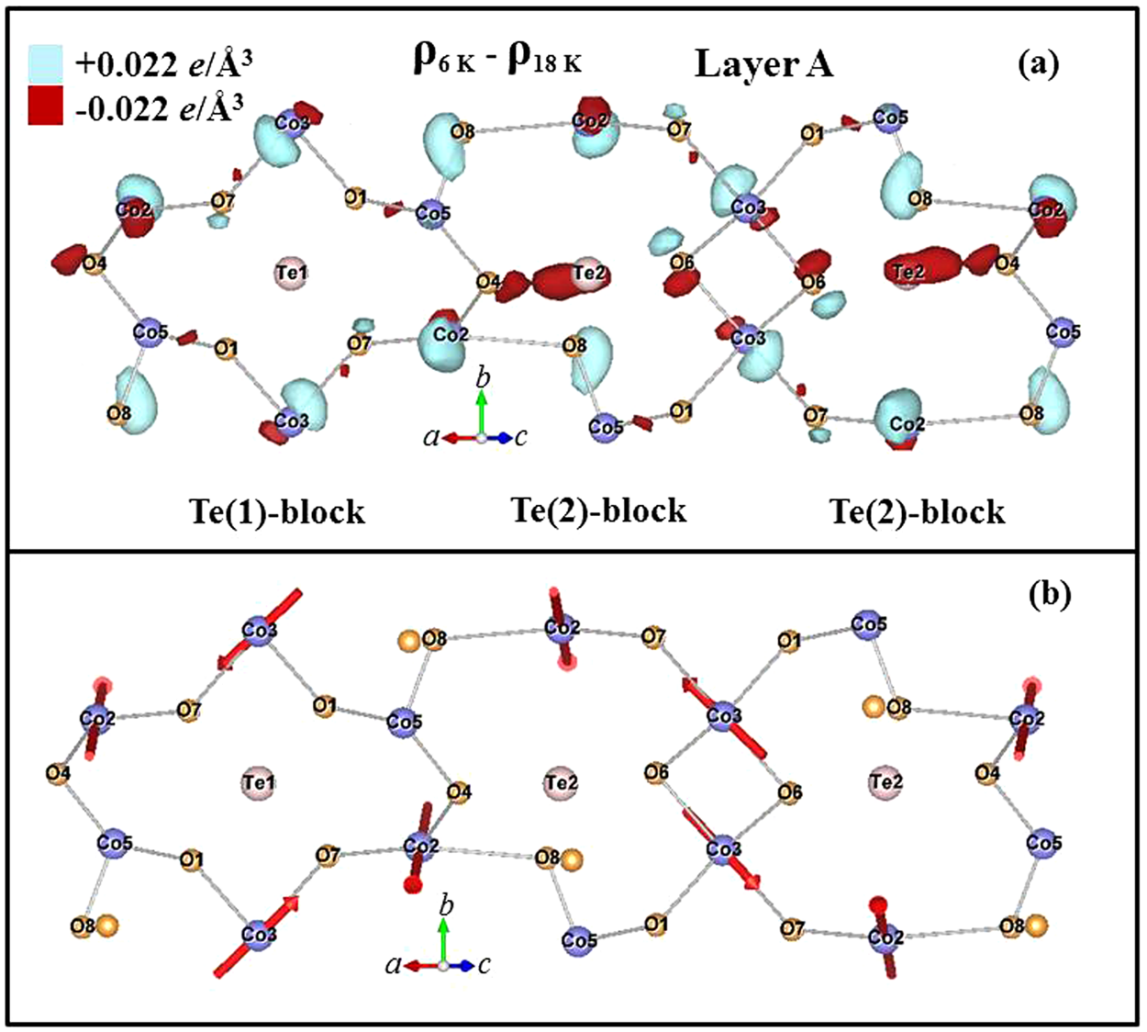
(**a**) Contour map of the electronic charge density in Layer A that develops upon cooling from 18 K to 6 K, as obtained from the x-ray diffraction data. The regions marked by contours for a difference ECD of + 0.022 *e*/Å^3^ are marked by light (turquoise) shading, while those of −0.022 *e*/Å^3^ are marked in dark (red) shading. (**b**) Arrows are used to indicate the electric dipoles that are associated with the difference electronic charge density on the ions, revealing an antiferroelectric-type of structure.

Synchrotron x-ray diffraction measurements reveal the interesting result that the ferroelectric transition is accompanied by significant electronic charge redistributions. The most pronounced changes occur around the Te(2) ions on the 8 *f* sites and their neighboring O(4) ions in Layer A, while the Te(1) ions on the 4*b* sites in Layer A contribute much less, perhaps none, to this behavior. Here, we adopt the labeling used in Table II of ref. 17 for the ions in the unit cell, where we have presented detailed crystallographic refinements of neutron powder diffraction data. Table 1 contains a typical complete refinement of the x-ray data at 40 K for comparison. These changes in the charge distributions are better revealed in the difference electronic charge density (ECD) plots, where the ECD distribution at 18 K is subtracted from that at 6 K. Such a difference ECD plot for the ions in Layer A is illustrated in Fig. 12a, where the contours for a difference ECD of +0.022 *e*/Å^3^ are marked by light (turquoise) shading while those of −0.022 *e*/Å^3^ are marked in dark (red) shading. The atomic positions with negative values represent the locations where the electronic charges are less at 6 K, being redistributed to the positions with positive values. These ECD maps were obtained by employing the General Structure Analysis System (GSAS) program, starting with profile refinements of the high-resolution x-ray diffraction patterns, followed by calculation of the inverse Fourier transforms of the structure factors to extract the electron density distribution. The observed and fitted x-ray diffraction patterns obtained at 6, 18, 21 and 30 K can be found in the Supplementary Information.

**Table 1:** Refinement of the x-ray data at 40 K. The refinements are in excellent agreement with the refined neutron diffraction data^17^.

Co_3_TeO_6_						
*Monoclinic C2*/*c*, (No. 15), T = 40 K,						
*a* = 14.7799(3) Å, *b* = 8.8303(2) Å, *c* = 10.3311(2) Å, *β* = 94.903(1)°						
Atom	x	y	Z	M	B_iso_(Å^2^)	Occupancy
Te1	0	0.5	0.5	4*b*	2.82(6)	0.99(1)
Te2	0.660(1)	−0.498(1)	0.299(1)	8*f*	3.35(5)	0.99(1)
Co1	0.5	−0.186(1)	0.25	4*e*	0.83(9)	1.01(1)
Co2	0.857(1)	−0.354(1)	0.232(1)	8*f*	0.52(9)	1.01(1)
Co3	0.522(1)	−0.652(1)	0.040(1)	8*f*	0.38(8)	1.00(1)
Co4	0.666(1)	−0.296(1)	0.056(1)	8*f*	0.70(9)	1.01(1)
Co5	0.797(1)	−0.361(1)	0.571(1)	8*f*	0.54(8)	0.99(2)
O1	0.926(1)	−0.323(2)	0.567(1)	8*f*	4.2(5)	0.99(2)
O2	0.591(1)	−0.333(2)	0.203(1)	8*f*	3.6(5)	0.99(2)
O3	0.593(1)	−0.648(1)	0.188(1)	8*f*	1.8(4)	1.02(3)
O4	0.752(1)	−0.521(2)	0.660(1)	8*f*	4.8(5)	0.98(3)
O5	0.933(1)	−0.504(2)	0.333(1)	8*f*	3.1(4)	1.00(1)
O6	0.582(1)	−0.522(1)	0.447(1)	8*f*	1.1(3)	1.01(2)
O7	0.918(1)	−0.662(1)	0.563(1)	8*f*	0.84(9)	0.97(2)
O8	0.736(1)	−0.329(2)	0.398(1)	8*f*	1.5(4)	1.00(3)
O9	0.732(1)	−0.664(2)	0.397(1)	8*f*	3.3(4)	0.99(2)
χ^2^ = 1.255, R_p_ = 3.51%, R_wp_ = 5.15%

**Figure 13:**
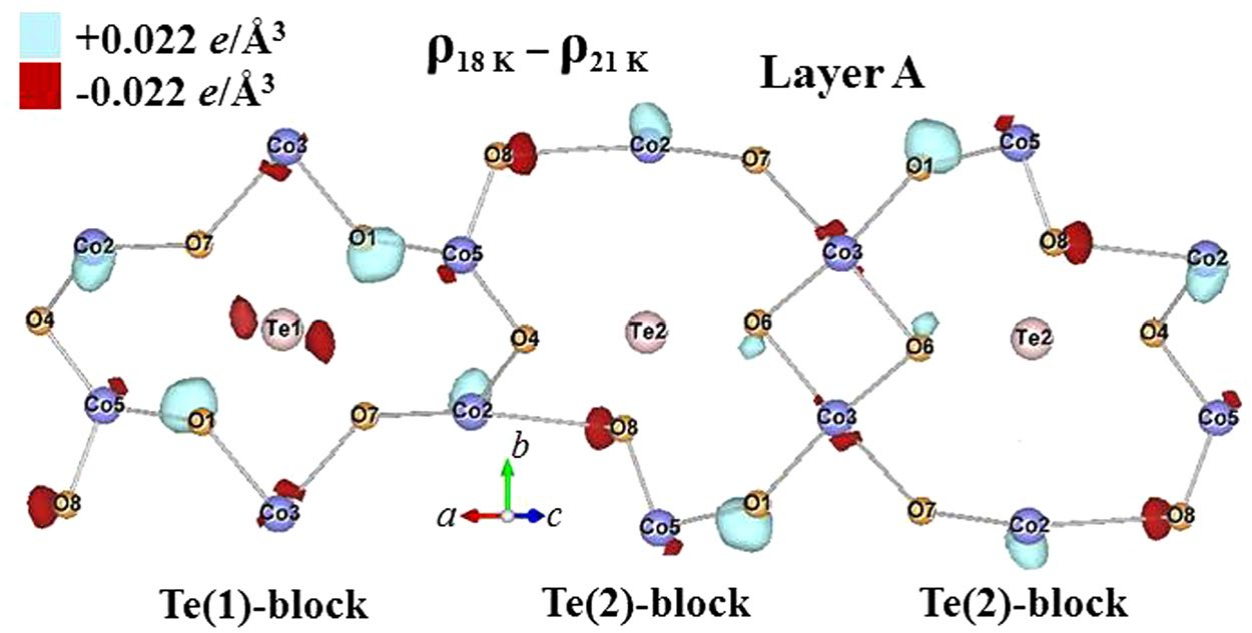
Contour map of the electronic charge density in Layer A that develops upon cooling from 21 K to 18 K, as obtained from the x-ray diffraction data. The regions marked by contours for a difference ECD of + 0.022 *e*/Å^3^ are marked by light (turquoise) shading, while those of −0.022 *e*/Å^3^ are marked in dark (red) shading.

**Figure 14:**
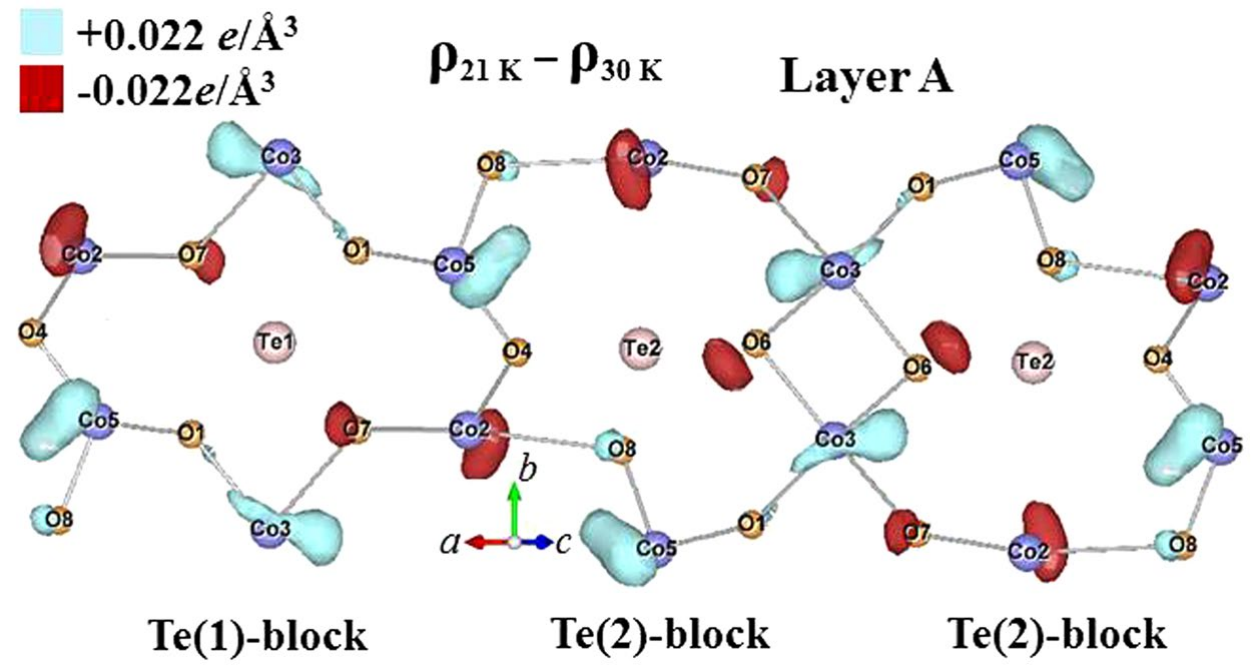
Contour map of the electronic charge density in Layer A that develops upon cooling from 30 K to 21 K, as obtained from the x-ray diffraction data. The regions marked by contours for a difference ECD of + 0.022 *e*/Å^3^ are marked by light (turquoise) shading, while those of −0.022 *e*/Å^3^ are marked in dark (red) shading.

There are three crystallographically distinct Co ions and two Te ions in Layer A. The loss of a significant amount of electronic charge around the Te(2)-O(4) bonding through the ferroelectric transition at 18 K is clearly revealed. In addition to a large amount of electronic charge shift to the neighboring Co(2)-O(8) bond, an increase of the electronic charge in the Co(3)-O(7) bonding and a loss of electronic charge in the Co(3)-O(6) bonding are noticeably evident. The changes in the electronic charges are strongly directional, and favor several specific crystallographic directions, rather than being evenly distributed around the atoms. Note that the electric polarization is also a result of strongly directional charge distribution. The observed charge redistribution along specific crystallographic directions could be an important source for the development of ferroelectricity. The Co ions in Layer A form two edge-sharing but crystallographically distinct honeycombs, one accommodating the Te(1) ion at the center [marked Te(1)-block], while the other accommodates the Te(2) ion [marked Te(2)-block], as illustrated in Fig. 12a. The two neighboring Co ions are interconnected through an O ion, forming a severely distorted Co-O honeycomb web. The geometric distortion of the Te(2)-block is much more severe than that of the Te(1)-block, and the crystalline symmetry of the Te(2)-blocks is also considerably lower. Interestingly, the supply of electronic charge from the Te-O ions to their neighboring ions through the ferroelectric transition occurs only in the severely distorted Te(2)-block, not in the crystallographically more symmetrical Te(1)-block. In addition, the crystalline symmetry of the Te(1)-block gives rise to an exact cancellation for the ECD in this local block, but a net difference ECD appears in the Te(2)-block, resulting from the difference in the changes in the ECD for the Co(2)-O(7) and for the Co(5)-O(1) bonds through the transition. The net difference ECD in the Te(2)-block can only be cancelled with that in the neighboring Te(2)-block, which is a large distance away [a separation of 6.604 Å between the two neighboring Te(2) ions]. Apparently, the Te(2)-blocks are connected more strongly to the development of ferroelectricity than the Te(1)-blocks. No such behavior was detected in Layer B. No noticeable amount of charge transfer from the Te(2) ions was found on cooling through T_M2_ from 21 to 18 K (Fig. 13) or through T_M1_ from 30 to 21 K (Fig. 14). Note that the difference ECD forms an electric dipole on the ion. The sizes of the electric dipoles on the Co(2) and Co(3) ions are particularly significant, as illustrated in Fig. 12b, showing that the ferroelectricity is mainly contributed from the Co(2) and Co(3) ions. It appears that these electric dipoles configure into structure that is primarily antiferroelectic in nature.

**Figure 15:**
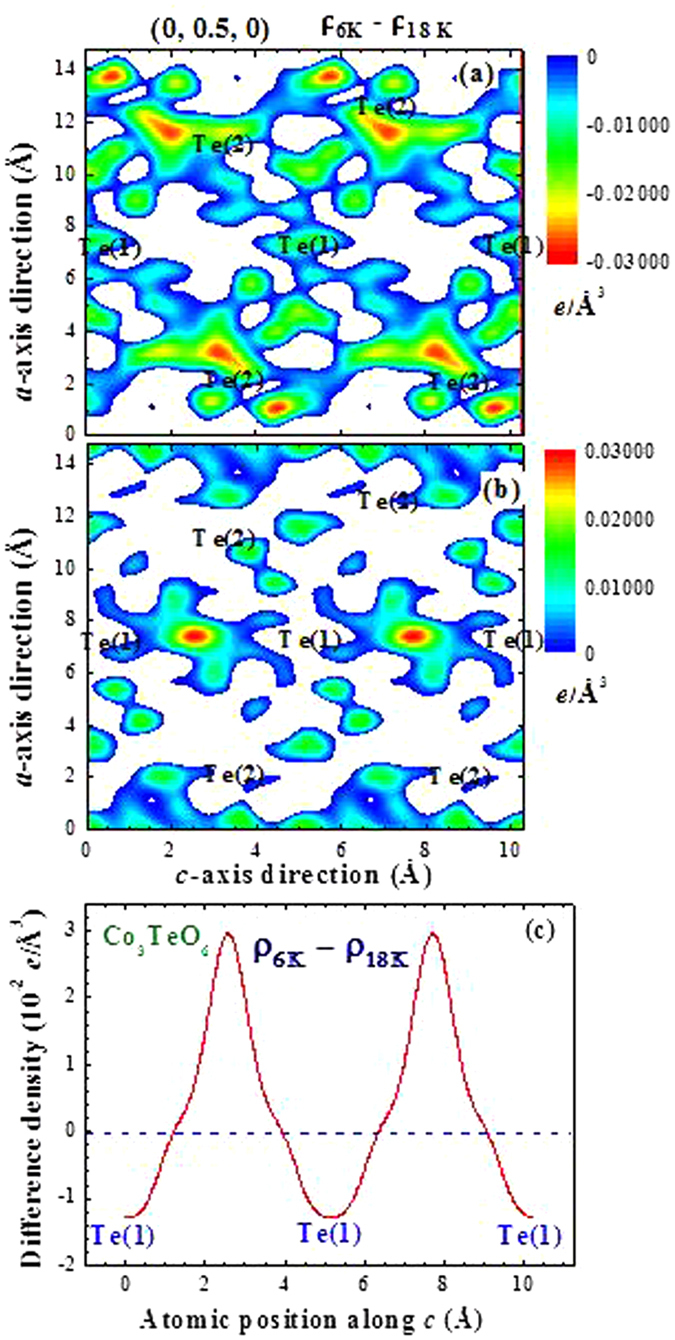
Portions of the difference electronic charge density maps in the y = 0.5 crystallographic plane, where (**a**) the electronic charge densities increase and (**b**) the electronic charge decrease, on cooling from 18 to 6 K. The color bars are in units of *e*/Å^3^. (**c**) Changes of the electronic charge density along the [001] crystallographic direction in the (0 0.5 0.835) plane upon cooling from 18 to 6 K.

Four separate layers (marked A, B, A′, B′) may be distinguished in a unit block of Co_3_TeO_6_, and the whole unit cell may be obtained by translating the unit block along the *a*-axis direction (Fig. 1). Layer A′ and Layer B′ can be reached by a translation operation of Layer A and Layer B, respectively, through (*a*/2 *b*/2 0). Redistribution of a considerable amount of electronic charge of the Te(1) ions through ferroelectric transition is also evident, as illustrated in Fig. 15a and b. This electron density contour map of the (0, 0.5, 0) crystallographic plane was obtained by slicing the electron density 0.025 Å below and above the plane. The electronic charge density along the $[\bar{1}0\bar{1}]$ crystallographic direction can be obtained by cutting the density map along that direction. There is a significant increase of electronic charges between the two neighboring Te(1) ions along the [001] direction upon cooling through the ferroelectric transition (Fig. 15b and c), revealing a better atomic connection between Layer A and Layer B in the ferroelectric state. These results clearly show that the Te(2) ions supply electronic charges to their neighboring ions while the electrons of the Te(1) ions become more widely distributed upon cooling through the ferroelectric transition. Apparently, both the Te(1) and Te(2) ions participate in the ferroelectric transition albeit in a different fashion.

## Conclusions

The present results go a long way in understanding the complicated nature of the magnetic orderings, coupling to the charge densities and ferroelectric order, and resolving some of the discrepancies in earlier work. Co_3_TeO_6_ initially develops magnetic order at T_M1_ = 26 K, which is fully incommensurate in nature in *h*, *k*, and *l*. In particular, there is no commensurate magnetic component to the order in the temperature range between T_M1_ and T_M2_. Moreover, we do not observe any other type of transition above T_M1_, in contrast to reports in powders of a transition at 34 K (see the discussion in ref. 19), nor do we observe any indication of ferromagnetic correlations in the paramagnetic state (T > T_M1_) in our neutron scattering data, in contrast to Singh, *et al*.^19^. Commensurate magnetic order develops separately at T_M2_ = 19.5 K in the Γ_4_ irreducible representation. With regard to the symmetries of the magnetic structures, we note that we have previously provided the irreducible representations for these commensurate structures^17^, while for the incommensurate order there is no subgroup symmetry analysis to discuss since they are incommensurate in all three components, and so group theory is not particularly enlightening. Between 19.5 K and 18 K the *h* component of the incommsurability splits into two peaks, one of which abruptly disappears below T_M3_ = 18 K where (weak) ferroelectricity develops, an additional commensurate magnetic structure consistent with Γ_3_ irreducible representation appears, and the *k* component of the incommensurability vanishes. Below T_M4_ = 15 K the k component of the incommensurability is re-established, and second-order magnetic satellite peaks develop indicating a small net magnetization has developed as indicated by Toledano, *et al*.^16^ and suggested by Singh, *et al*.^19^. Finally, we find no commensurate intensities from the (01/21/4)-type of reflections that were proposed in ref. 9 on the basis of powder diffraction data and were the basis of a symmetry analysis in ref. 16.

Synchrotron powder x-ray diffraction measurements show that Te ions participate in the development of ferroelectricity. There are two crystallographically distinct Te ions in monoclinic Co_3_TeO_6_. The Te ions located at the center positions of the severely distorted Co honeycomb contribute significantly to the development of ferroelectricity by supplying a significant electronic charge to their neighboring Co and O ions, but the changes are unevenly distributed throughout the surrounding honeycomb structure. No such behavior is found in the Te ions located at the center positions of the other Co honeycombs with a higher degree of crystalline symmetry, although the electronic charges of these Te ions become more widely distributed toward the neighboring layers through ferroelectric transition. We demonstrate that the development of ferroelectricity in Co_3_TeO_6_ is closely linked to the increase of electronic charges in specific regions, where local crystallographic distortions and/or asymmetry appear. This spontaneous polarization appears to be associated with a charge redistribution that is primarily antiferroelectric in nature. This antiferroelectric behavior is consistent with the unusual anomalies in the lattice parameters at the ferroelectric transition, where there is sizable negative thermal expansion in all three lattice parameters^17^. Anomalies have been observed in other ferroelectrics such as HoMn_2_O_5_, DyMn_2_O_5_, and TbMn_2_O_5_^23^, TbMnO_3_^33^, GdMnO_3_^34^, Ni_3_V_2_O_8_^35^, and even PbTiO_3_^36^, but with mixed behavior of expansion and contraction.

## Electronic supplementary material

Supplementary Information
