# Supplementary material for: Complex magnetic incommensurability and electronic charge transfer through the ferroelectric transition in multiferroic Co3TeO6
Source: Sci Rep. 2017 Jul 25;7:6437. doi: 10.1038/s41598-017-06651-9 (PMC5527072; doi:10.1038/s41598-017-06651-9)
Supplement: Supplementary file 1 — Supplementary Information [file 41598_2017_6651_MOESM1_ESM.pdf]

Supplementary Information

Complex magnetic incommensurability and electronic charge transfer through the ferroelectric transition in multiferroic Co_3TeO_6

Chi-Hung Lee,[1] Chin-Wei Wang,[2] Yang Zhao,[3,4] Wen-Hsien Li,[1,*] Jeffrey W. Lynn,[3] A. Brooks Harris,[5] Kirrily Rule,[6] Hung-Duen Yang,[7] and Helmuth Berger[8]

[1]Department of Physics, National Central University, Jhongli 32001, Taiwan
[2]Neutron Group, National Synchrotron Radiation Research Center, Hsinchu 30076, Taiwan
[3]NIST Center for Neutron Research, National Institute of Standards and Technology, Gaithersburg, Maryland 20899, USA
[4]Department of Materials Science and Engineering, University of Maryland, College Park, MD 20742 USA
[5]Department of Physics and Astronomy, University of Pennsylvania, Philadelphia, PA 19104 USA
[6]Bragg Institute, Australian Nuclear Science and Technology Organisation, Lucas Heights, NSW 2234, Australia
[7]Department of Physics and Center for Nanoscience and Nanotechnology, National Sun Yat-Sen University, Kaohsiung 80424, Taiwan
[8]Institute of Physics of Complex Matter, EPFL, Lausanne, Switzerland

Sample fabrication. Single crystals of Co_3TeO_6 were synthesized via chemical vapor transport redox reactions.[S1] The Co_3O_4, TeO_2, and $CoCl_2$ powders were mixed thoroughly using a molar ratio of 4:3:1 before being loaded into one end of a silica tube. The tube was then evacuated to 10^{-5} torr, and filled with HCl gas that acts as the transporting agent, before being sealed off. The ampoule was subsequently placed in a two-zone furnace, with the temperatures of the charge and growth zones set to 973 and 873 K, respectively. This allowed the transportation of the starting materials from the charge zone to epitaxially grow into single crystals in the growth zone. The resultant single crystals were dark-violet in color. The single crystal used in the present measurements weighed 101 mg, with a size of 14.1×2.2×0.9 mm^3. A number of small crystals were crushed into powder for x-ray and neutron powder diffraction measurements.

Sample characterization. X-ray diffraction was first used to check the powdered sample. No obvious differences were found in the x-ray diffraction patterns taken from different portions of the powdered sample. The high-resolution neutron and X-ray diffraction patterns were analyzed using the General Structure Analysis System (GSAS) program[S2]

following the Rietveld profile refining method. Figure S1 shows the observed (crosses) and calculated (solid lines) neutron patterns, collected at 30 K, assuming the same monoclinic *C*2/*c* (No. 15, *b* unique) symmetry reported[S1,S3] for the compound at room temperature. They agree very well, judging from the difference pattern plotted at the bottom and the goodness parameters listed in Table S1. The crystalline parameters thus obtained for the present Co_3TeO_6 at 30 K are summarized in Table S1. No site deficiency is found. The crystalline unit cell contains 12 chemical units of Co_3TeO_6, with five Co sites, two Te sites, and nine O sites in the unit cell that are crystallographically distinguishable. There is no structural change or lattice distortion that can be identified from the high-resolution X-ray or neutron diffraction patterns between 7 and 300 K.

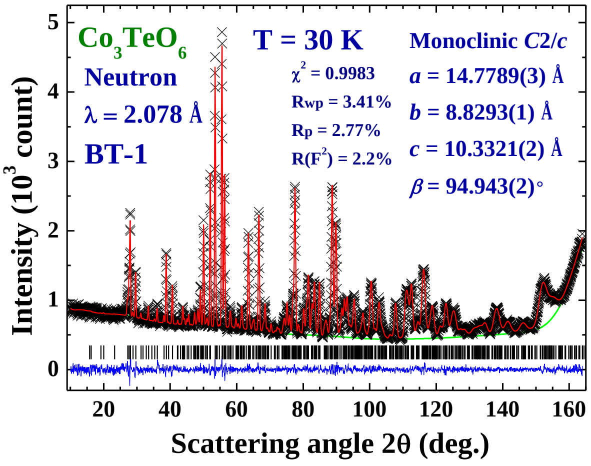

Figure S1. Observed (crosses) and fitted (solid lines) high-resolution neutron powder diffraction pattern at 30 K, assuming a monoclinic symmetry of the space group *C2/c*. The differences between the calculated and observed patterns are plotted at the bottom. The solid vertical lines mark the calculated positions of the Bragg reflections of the proposed crystalline structure.

Magnetic susceptibility. Figure S2a shows the temperature dependence of the in-phase component of the ac magnetic susceptibility $\chi'(T)$ of the powdered sample, measured without the presence of magnetic field. In the high temperature regime the thermal profile of the magnetic susceptibility χ' can be described by the Curie-Weiss behavior (solid line in Fig. S2b). The $\chi'(T)$ departs from the Curie-Weiss profile below 60 K, indicating the appearance of magnetic correlations. However, this small amount of magnetic correlations is beyond the detections of the neutron diffraction at current resolution. There is no essential differences can be identified between the diffraction intensities taken at 30 and 80 K. A downturn in $\chi'(T)$ appears at 30 K, with an abrupt drop at 26 K. Another anomaly appears at 18 K, where a change of the thermal reduction rate of χ' is evident. In addition to the Curie-Weiss behavior departs below 60 K, the T_{M1} = 26 K and T_{M3} = 18 K identified

using neutron diffraction are also revealed in the χ'(T) curve taken without the presence of an applied magnetic field. T_{M1} and T_{M3} are also visible in the iso-field thermal magnetization M(T) curve (Fig. S2c) taken at H_a = 10 kOe, revealing a net magnetization of 1.29 emu/g for the Co_3TeO_6 at 1.8 K.

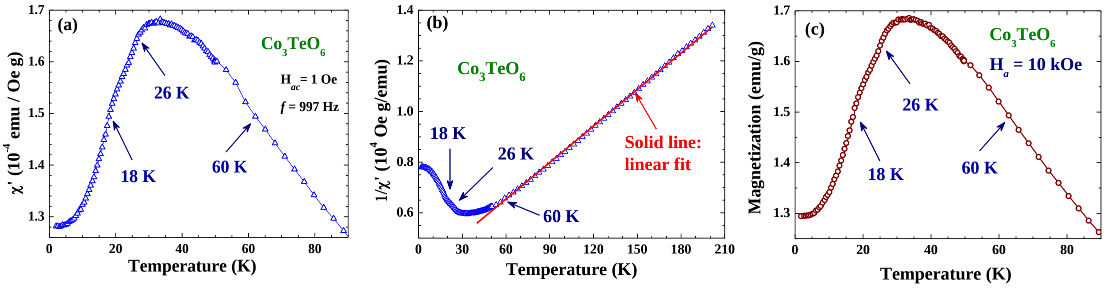

Figure S2. (a) χ'(T) curve of powdered Co_3TeO_6, measured using a driving ac magnetic field with a root-mean-square strength of 1 Oe and a frequency of 997 Hz. (b) Temperature dependence of 1/χ' , where the solid line indicates the results of a linear fit to the data at high temperatures. (c) M(T) curve of powdered Co_3TeO_6, measured at an applied magnetic field of 10 kOe.

Projections of ICM satellite reflections. Four ICM satellite components associated with each Bragg peak are seen for the reflections in the (*hk0*) and (*0kl*) scattering planes; whereas only two ICM components associated with each Bragg peak revealed in the (*h0l*) scattering plane with the modulation only in the $+q_h$ or in the $+q_l$ component. As illustrated in Fig. S3a the four ICM reflections at ($+q_h$, $2+q_k$, $-q_l$), ($+q_h$, $2-q_k$, $-q_l$), ($-q_h$, $2+q_k$, $+q_l$) and ($-q_h$, $2-q_k$, $+q_l$) (crosses) will be detected by the instrumental resolution at four positions (solid circles) upon scanning in the (*hk0*) scattering plane, but will be detected at only two positions when is scanning in the (*h0l*) scattering plane.

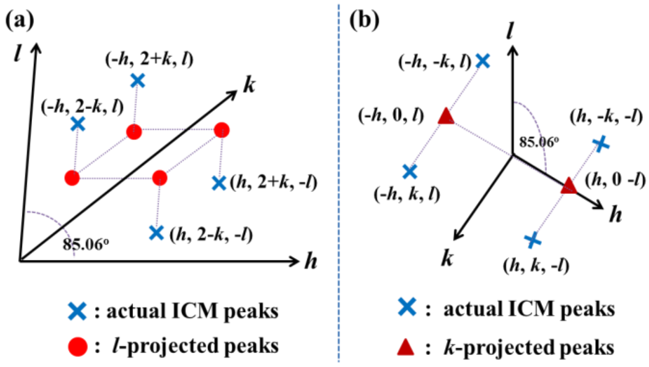

Figure S3. Schematic diagram of the projection scan of a set of ICM peaks at (*hkl*)+$\boldsymbol{q_i}$, where *h*, *k* and *l* are integers and $\boldsymbol{q_1}$ = (q_h, q_k, q_l), $\boldsymbol{q_2}$ = (q_h, $-q_k$, q_l), $\boldsymbol{q_3}$ = ($-q_h$, q_k, $-q_l$) and $\boldsymbol{q_4}$ = ($-q_h$, $-q_k$, $-q_l$). In (a) we show the projection into the (*hk0*) plane, which results from the experimental situation in which the observed spectrum at l = 0 is essentially an integration over *l*. (b) is similar to (a) except that the projection is unto the (*h0l*) plane, in which case there are only two projected reflections.

Low temperature X-ray diffraction patterns. The high-resolution X-ray diffraction patterns, obtained from a synchrotron source, of the powdered sample at 6, 18, 21 and 30 K are shown in Figs. S4a, S4b, S4c and S4d, respectively. These diffraction patterns were analyzed using the General Structure Analysis System (GSAS) program[S2] following the Rietveld profile refining method. The crystalline parameters thus obtained at 6, 18, 21 and 30 K are summarized in Tables S2, S3, S4 and S5, respectively. These patterns contain diffraction intensities from atomic electrons but not from the magnetic component. They may be used to extract the electronic charge distribution by profile refinements of the X-ray diffraction patterns, followed by calculation of the inverse Fourier transforms of the structure factors. The lattice constants along all three crystallographic directions at 18 K are slightly but noticeably smaller than those at 6 K, revealing a negative thermal expansion for the Co_3TeO_6 in this temperature regime. Changes of the relative intensities, hence the relative atomic positions, are also revealed on cooling from 18 to 6 K (Fig. S5), reflecting a change of the electronic charge distribution on cooing from 18 to 6 K.

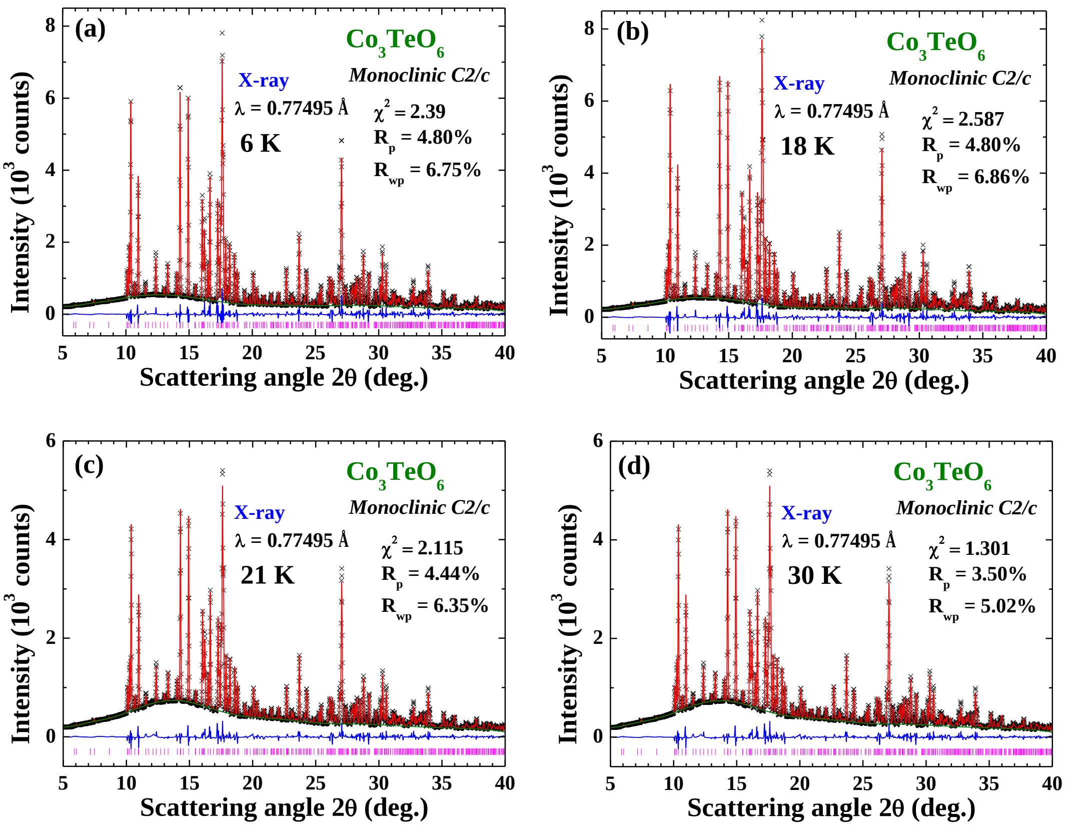

Figure S4. Observed (crosses) and fitted (solid lines) high-resolution X-ray powder diffraction pattern at (a) 6 K, (b) 18 K, (c) 21 K and (d) 30 K, assuming a monoclinic symmetry of the space group *C2/c*. The differences between the calculated and observed patterns are plotted at the bottom.

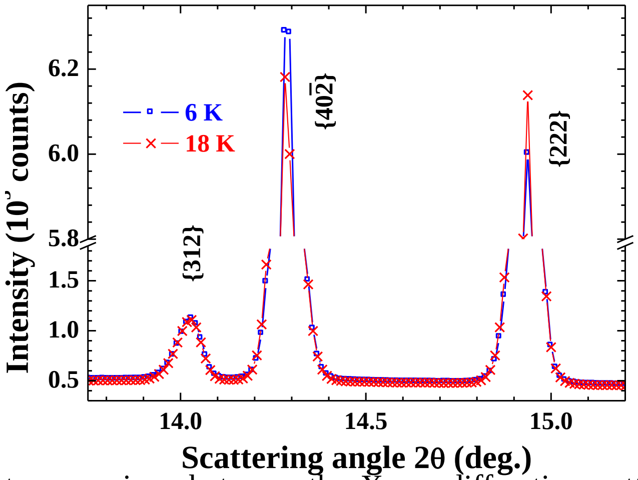

Figure S5. A direct comparison between the X-ray diffraction patterns obtained at 6 K (open squares) and 18 K (crosses), revealing noticeable changes of the relative intensities.

References

S1. Becker, R., Johnssona, M. & Berger, H. A new synthetic cobalt tellurate: Co_3TeO_6. *Acta Cryst.* C**62**, i67-i69 (2006).

S2. Larson, A. C. & von Dreele, R. B., General Structure Analysis System Report LA-UR-86-748 (Los Alamos, NM: Los Alamos National Laboratory) (2004).

S3. Hudl, M., Mathieu, R., Ivanov, S. A., Weil, M., Carolus, V., Lottermoser, Th., Fiebig, M., Tokunaga, Y., Taguchi, Y., Tokura, T. & Nordblad, P. Complex magnetism and magnetic-field-driven electrical polarization of Co_3TeO_6. *Phys. Rev. B* **84** 180404(R) (2011).

Table S1. Refined lattice parameters and atomic positions of Co_3TeO_6 at 30 K, assuming a symmetry of space group *C*2/*c* (Z=12).

**Co_3TeO_6**<br>Monoclinic *C2*/*c*, (No. 15), T = 30 K, Neutron<br>a = 14.7789(3) Å , b = 8.8293(1) Å , c = 10.3321(2) Å , β = 94.905(2)^o						
Atom	x	y	Z	M	B_{iso}(Å ^{2})	Occupancy
Te(1)	0	0.5	0.5	4*b*	0.65(9)	0.99(2)
Te(2)	0.6608(6)	-0.5052(18)	0.3008(8)	8*f*	0.57(7)	0.99(1)
Co(1)	0.5	-0.1760(37)	0.25	4*e*	0.18(9)	1.00(3)
Co(2)	0.8661(11)	-0.3577(29)	0.2285(17)	8*f*	1.3(3)	1.01(2)
Co(3)	0.5149(12)	-0.6585(29)	0.0500(14)	8*f*	0.9(1)	1.01(3)
Co(4)	0.6695(17)	-0.2861(19)	0.0586(22)	8*f*	0.70(9)	1.01(3)
Co(5)	0.7943(14)	-0.3571(27)	0.5666(18)	8*f*	1.1(2)	1.00(3)
O(1)	0.9281(7)	-0.3336(12)	0.5569(10)	8*f*	1.0(1)	0.99(2)
O(2)	0.5940(7)	-0.3490(13)	0.2076(10)	8*f*	0.53(9)	0.99(1)
O(3)	0.6011(7)	-0.6544(14)	0.1948(11)	8*f*	0.94(9)	1.02(1)
O(4)	0.7504(5)	-0.5269(12)	0.6696(7)	8*f*	1.1(1)	1.00(3)
O(5)	0.9272(5)	-0.5144(14)	0.3365(7)	8*f*	0.88(8)	1.00(1)
O(6)	0.5843(5)	-0.5120(14)	0.4380(8)	8*f*	0.82(8)	1.01(1)
O(7)	0.9275(7)	-0.6590(12)	0.5673(10)	8*f*	0.84(9)	0.98(1)
O(8)	0.7372(8)	-0.3500(14)	0.3964(10)	8*f*	0.63(9)	1.01(1)
O(9)	0.7285(7)	-0.6664(14)	0.3884(10)	8*f*	1.5(1)	0.99(2)
χ^2 = 0.9983, R_p= 2.77%, R_{wp}= 3.41%						

Table S2. Refined lattice parameters and atomic positions of Co_3TeO_6 at 6 K, assuming a symmetry of space group *C*2/*c* (Z=12).

Atom	x	y	z	M	B_{iso}(Å ^{2})	Occupancy
**Co_3TeO_6**						
Monoclinic *C2*/*c*, (No. 15), T = 6 K, synchrotron X-ray						
a = 14.7803(3) Å , *b* = 8.8305(2) Å , *c* = 10.3314(2) Å , *β* = 94.903(1)^o						
Te1	0	0.5	0.5	4*b*	2.88(6)	0.99(1)
Te2	0.660(1)	-0.499(1)	0.299(1)	8*f*	2.64(5)	0.99(1)
Co1	0.5	-0.186(1)	0.25	4*e*	0.49(9)	1.01(1)
Co2	0.857(1)	-0.354(1)	0.231(1)	8*f*	0.44(9)	1.01(1)
Co3	0.522(1)	-0.652(1)	0.040(1)	8*f*	0.11(1)	1.00(1)
Co4	0.666(1)	-0.296(1)	0.056(1)	8*f*	0.74(9)	1.01(1)
Co5	0.797(1)	-0.361(1)	0.571(1)	8*f*	0.51(8)	0.99(2)
O1	0.925(1)	-0.323(2)	0.565(1)	8*f*	3.6(5)	0.99(2)
O2	0.590(1)	-0.327(2)	0.206(1)	8*f*	3.5(5)	0.99(2)
O3	0.593(1)	-0.647(1)	0.189(1)	8*f*	1.7(4)	1.02(3)
O4	0.755(1)	-0.520(2)	0.660(1)	8*f*	4.9(5)	0.99(3)
O5	0.931(1)	-0.506(2)	0.332(1)	8*f*	2.7(4)	1.00(1)
O6	0.582(1)	-0.523(1)	0.447(1)	8*f*	0.4(1)	1.01(2)
O7	0.919(1)	-0.664(1)	0.564(1)	8*f*	0.47(9)	0.97(2)
O8	0.735(1)	-0.328(2)	0.399(1)	8*f*	1.3(4)	1.00(3)
O9	0.730(1)	-0.663(2)	0.397(1)	8*f*	3.3(4)	0.99(2)
χ^2 = 2.390, R_p= 4.80%, R_{wp}= 6.75%						

Table S3. Refined lattice parameters and atomic positions of Co_3TeO_6 at 18 K, assuming a symmetry of space group *C*2/*c* (Z=12).

**Co_3TeO_6**						
Monoclinic *C2*/*c*, (No. 15), T = 18 K, synchrotron X-ray						
a = 14.7779(3) Å , *b* = 8.8292(2) Å , *c* = 10.3295(2) Å , *β* = 94.901(1)^{o}						
Atom	x	y	Z	M	B_{iso}(Å ^{2})	Occupancy
Te1	0	0.5	0.5	4*b*	2.87(6)	0.99(1)
Te2	0.660(1)	-0.498(1)	0.299(1)	8*f*	2.63(5)	0.99(1)
Co1	0.5	-0.186(1)	0.25	4*e*	0.39(9)	1.01(1)
Co2	0.857(1)	-0.354(1)	0.232(1)	8*f*	0.44(9)	1.01(1)
Co3	0.522(1)	-0.652(1)	0.040(1)	8*f*	0.11(1)	1.00(1)
Co4	0.666(1)	-0.296(1)	0.056(1)	8*f*	0.70(9)	1.01(1)
Co5	0.797(1)	-0.361(1)	0.571(1)	8*f*	0.50(8)	0.99(2)
O1	0.924(1)	-0.324(2)	0.565(1)	8*f*	3.5(5)	0.99(2)
O2	0.590(1)	-0.324(2)	0.206(1)	8*f*	3.5(5)	0.99(2)
O3	0.594(1)	-0.647(1)	0.188(1)	8*f*	1.6(4)	1.02(3)
O4	0.754(1)	-0.521(2)	0.662(1)	8*f*	4.9(5)	0.99(3)
O5	0.931(1)	-0.507(2)	0.333(1)	8*f*	2.7(4)	1.00(1)
O6	0.582(1)	-0.522(1)	0.447(1)	8*f*	0.3(1)	1.01(2)
O7	0.919(1)	-0.666(1)	0.564(1)	8*f*	0.45(9)	0.97(2)
O8	0.736(1)	-0.328(2)	0.397(1)	8*f*	1.3(4)	1.00(3)
O9	0.732(1)	-0.665(2)	0.396(1)	8*f*	3.1(4)	0.99(2)
χ^2 = 2.587, R_p= 4.80%, R_{wp}= 6.86%						

Table S4. Refined lattice parameters and atomic positions of Co_3TeO_6 at 21 K, assuming a symmetry of space group *C*2/*c* (Z=12).

**Co_3TeO_6**						
Monoclinic *C2*/*c*, (No. 15), T = 21 K, synchrotron X-ray						
a = 14.7791(3) Å , b = 8.8301(2) Å , c = 10.3308(2) Å , β = 94.904(1)^o						
Atom	x	y	Z	M	B_{iso}(Å ^{2})	Occupancy
Te1	0	0.5	0.5	4*b*	2.89(6)	0.98(1)
Te2	0.661(1)	-0.499(1)	0.299(1)	8*f*	2.65(5)	0.99(1)
Co1	0.5	-0.186(1)	0.25	4*e*	0.43(9)	1.01(1)
Co2	0.857(1)	-0.354(1)	0.232(1)	8*f*	0.45(9)	1.01(1)
Co3	0.522(1)	-0.652(1)	0.040(1)	8*f*	0.10(1)	0.99(1)
Co4	0.666(1)	-0.296(1)	0.056(1)	8*f*	0.73(9)	1.01(1)
Co5	0.798(1)	-0.362(1)	0.571(1)	8*f*	0.53(8)	1.00(2)
O1	0.925(1)	-0.323(2)	0.567(1)	8*f*	3.6(5)	0.99(2)
O2	0.591(1)	-0.325(2)	0.204(1)	8*f*	3.9(5)	0.99(2)
O3	0.593(1)	-0.646(1)	0.188(1)	8*f*	1.5(4)	1.02(2)
O4	0.752(1)	-0.520(2)	0.661(1)	8*f*	3.5(5)	0.99(3)
O5	0.932(1)	-0.506(2)	0.332(1)	8*f*	2.8(4)	1.00(1)
O6	0.582(1)	-0.523(1)	0.447(1)	8*f*	0.4(1)	1.01(2)
O7	0.919(1)	-0.666(1)	0.565(1)	8*f*	0.46(9)	0.98(2)
O8	0.736(1)	-0.328(2)	0.397(1)	8*f*	1.2(4)	1.00(3)
O9	0.732(1)	-0.665(2)	0.397(1)	8*f*	3.0(4)	0.99(2)
χ^2 = 2.115, R_p= 4.44%, R_{wp}= 6.35%						

Table S5. Refined lattice parameters and atomic positions of Co_3TeO_6 at 30 K, assuming a symmetry of space group *C*2/*c* (Z=12).

**Co_3TeO_6**<br>Monoclinic *C2*/*c*, (No. 15), T = 30 K, synchrotron X-ray<br>a = 14.7789(3) Å , b = 8.8302(2) Å , c = 10.3309(2) Å , β = 94.910(1)^o						
Atom	x	y	Z	M	B_{iso}(Å ^{2})	Occupancy
Te1	0	0.5	0.5	4*b*	2.99(6)	0.99(1)
Te2	0.660(1)	-0.499(1)	0.299(1)	8*f*	2.75(5)	0.99(1)
Co1	0.5	-0.186(1)	0.25	4*e*	0.49(9)	1.01(1)
Co2	0.858(1)	-0.354(1)	0.232(1)	8*f*	0.55(9)	1.01(1)
Co3	0.523(1)	-0.653(1)	0.041(1)	8*f*	0.19(1)	1.00(1)
Co4	0.666(1)	-0.296(1)	0.056(1)	8*f*	0.70(9)	1.00(1)
Co5	0.798(1)	-0.362(1)	0.571(1)	8*f*	0.63(8)	0.99(2)
O1	0.927(1)	-0.324(2)	0.568(1)	8*f*	3.9(5)	0.99(2)
O2	0.591(1)	-0.325(2)	0.206(1)	8*f*	3.8(5)	1.00(2)
O3	0.593(1)	-0.646(1)	0.188(1)	8*f*	1.9(4)	1.02(3)
O4	0.753(1)	-0.521(2)	0.661(1)	8*f*	3.9(5)	0.99(2)
O5	0.933(1)	-0.504(2)	0.332(1)	8*f*	3.8(4)	1.00(1)
O6	0.582(1)	-0.523(1)	0.447(1)	8*f*	0.5(1)	1.01(2)
O7	0.919(1)	-0.666(1)	0.564(1)	8*f*	0.66(9)	0.97(3)
O8	0.737(1)	-0.328(2)	0.397(1)	8*f*	1.9(4)	1.00(3)
O9	0.733(1)	-0.666(2)	0.397(1)	8*f*	3.9(4)	0.99(2)
χ^2 = 1.301, R_p= 3.50%, R_{wp}= 5.02%						
